# Effects of a Clinical Simulation-Based Training Program for Nursing Students to Address Social Isolation and Loneliness in the Elderly: A Quasi-Experimental Study

**DOI:** 10.3390/healthcare11182587

**Published:** 2023-09-19

**Authors:** María Jesús Hernández-López, María Ruzafa-Martínez, César Leal-Costa, Antonio Jesús Ramos-Morcillo, Isidora Díaz-García, María Verónica López-Pérez, Solanger Hernández-Méndez, Jessica García-González

**Affiliations:** 1 Faculty of Social and Health Sciences, University of Murcia, Av de las Fuerzas Armadas, 30800 Lorca, Spain; mariajesus.hernandez2@um.es (M.J.H.-L.); isidora.diaz@um.es (I.D.-G.); mariaveronica.lopez@um.es (M.V.L.-P.); solanger.hernandez@um.es (S.H.-M.); 2 Faculty of Nursing, University of Murcia, Av. Buenavista, 32, El Palmar, 30120 Murcia, Spain; maruzafa@um.es (M.R.-M.); ajramos@um.es (A.J.R.-M.); 3 Faculty of Health Sciences, University of Almeria, Carr. Sacramento, s/n, La Cañada, 04120 Almería, Spain; jgg145@ual.es

**Keywords:** nursing education, high-fidelity simulation training, aged, social isolation, loneliness

## Abstract

Introduction: The population of older adults is rapidly increasing worldwide, presenting both prospects and complexities for society and healthcare professionals to maximize the functional capacity of this age group. Social isolation and loneliness significantly affect this population. The objective was to determine the effectiveness, satisfaction, and perceptions of the simulation-based education practices of a training program for nursing students, which was created to palliate the social isolation and loneliness of older adults. Method: A quasi-experimental study was conducted with nursing students who participated in an online training program using teleservice based on high-fidelity clinical simulation. The program included asynchronous theoretical training and synchronous practical training using an online platform. Five scenarios were designed using simulated phone calls to address the social isolation and loneliness of older adults. Results: Twenty-five nursing students participated in the program, and they had a mean age of 27.44, with 76% of them being women. After the training program, the participants showed statistically significant improvements (*p* < 0.05) with respect to their knowledge and attitudes towards older adults, and the program was adapted to the best educational practices in simulations. Conclusions: Simulation-based online training efficiently improved the knowledge and attitudes of nursing students towards older adults, improving their ability to address social isolation and loneliness. The high satisfaction and adhesion to the best educational practices underline the usefulness of high-fidelity online simulations, especially in situations in which face-to-face training is not feasible, and accessibility and equilibrium could be guaranteed between work and personal life.

## 1. Introduction

The global elderly demographic is expanding at a swift pace, presenting both prospects and complexities for society and healthcare professionals with respect to optimizing the functional capabilities of this age group [1].

Social isolation and loneliness are two important problems of public health that strongly affect this population group. Social isolation, understood as “the dissociation of social relations, connections with institutions or community participation” [2] is associated with aging and the belief of having health problems [3]. Physical fragility, as well as the death of family members and friends, contributes to these issues [4]. Also, social isolation and loneliness are predictors of mortality, which increases in older adults who are more socially isolated [5], and they are comparable to risk factors, such as tobacco use, and contribute even more to high blood pressure [6]. In contrast, social participation is an indicator of successful aging and quality of life [7], and it is associated with protective factors for human health, increasing the level of physical activity and cognitive functions [8].

However, living alone, being socially isolated, and feeling lonely, are different conditions, and even if there is a causal association, experiencing one of the conditions does not mean experiencing the others [4].

Loneliness is defined as “a subjective and negative feeling” [9] that accompanies perceived social isolation [10]. It is associated with living with a smaller number of people [3]; living in rural areas and small cities; being older; having a low level of education, low income, and bad health, with illnesses (81%), the death of a partner (79%), and lack of friends (67%) being the most common subjective causes [11]. Also, the death rate increases by 26% for people who are lonely [12], and it is a predictor of dementia, with both social isolation and loneliness being risk factors for cognitive deterioration [6]. Ultimately, the isolated older adult population utilizes more health resources; exhibits a greater risk of falls, more hospital admittances, and more institutionalization; and needs more at-home care [12].

The COVID-19 disease, due to SARS-CoV-2, disproportionately affected older adults, with higher mortality rates observed in this population [13]. Beyond the mortality data, the impact of the social restrictions associated with this pandemic, especially observed in older adults, must be underlined, as they provoked an increase in the social isolation of those older than 65 years of age [12,14,15]. Likewise, during the pandemic, an increase in loneliness from 37% to 51% was observed [16,17], with negative results in the well-being and health of older adults [18] who mentioned feeling more desperate (34.8%) and more depressed (44.8%) [19].

Given this background, it is necessary to propose interventions to improve the situation of isolation and loneliness relative to older adults, with the expectation that they will have a positive impact with respect to their lifestyle, health, and quality of life and at the same time a decrease in the number of adverse events and the need for health resources. To design these interventions, two important aspects must be considered. On the one hand, the responsibility for taking care of these matters has mainly fallen on civil society, social services, and citizen associations, and in general terms, they are not part of the healthcare priorities of the Spanish health system [12]. The second aspect to consider is that the pandemic made the implementation of telemedicine via teleconsultations mandatory, and these are performed through telephone or video calls [20].

The “distance” interventions applied up to the present day have been classified into five categories: (1) videoconferences; (2) telephone calls; (3) online discussion groups/forums; (4) use of social networks; (5) interventions with multiple tools [21]. Many studies have demonstrated that some of these interventions decrease the levels of depression; improve support and social interaction; and reduce loneliness [22,23,24,25].

Presently, there is no doubt that clinical simulation is an essential component of health science education [26]. Clinical simulation plays a fundamental role in the training of health sciences students for the care of older adults [27,28,29]. It also provides opportunities for students to develop comprehensive care that is centered on the individual and the challenges faced by older adults. When practicing in a safe environment, students can improve their skills and, ultimately, the patient’s results, promote social connections, and reduce loneliness among older adults [30,31].

The existing data show the importance of addressing the problem of the social isolation and loneliness of older adults given their provoked increase due to the pandemic, the repercussions on physical and mental health, and the increase in the demand for health system services. In Spain, there are currently no measures or social policies implemented to help prevent them, and the evidence on the prevention of isolation is still scarce [32]. There is therefore a current need to tend to the elderly who suffer from social isolation and loneliness using the application of effective and innovative measures that imply the use of resources, such as telemedicine and the involvement of health and social agents from different areas.

Our study is framed within the first phase of the telehealth program “HELPeN”, directed to older adults in social isolation who live in the community. This telecare program comprises a telephone-based intervention led by nurses specializing in community health, geriatrics, and mental health. These expert nurses serve as mentors and supervisors for volunteer nursing students. The program was executed using a series of telephone calls, during which a comprehensive health and quality-of-life assessment will be conducted. Individualized care plans and interventions were implemented, and elderly individuals were continuously monitored for a duration of 9 months. We anticipate that this intervention positively impacted the lifestyle, health, and overall quality of life of the elderly population. Furthermore, it is expected to reduce feelings of loneliness and social isolation, consequently enhancing their quality of life. This, in turn, is anticipated to lead to a reduction in adverse events and a decreased demand for healthcare resources.

The objective of the present study was to determine the effectiveness, satisfaction, and perceptions of the simulation-based education practices of an online training program for nursing students created to palliate the social isolation and loneliness of older adults.

## 2. Materials and Methods

### 2.1. Design

A quasi-experimental study with pre–post measurements was conducted with a non-randomized intervention group composed of nursing degree students who participated in a high-fidelity clinical simulation (HFCS) telecare online training program in order to address social isolation and loneliness in older adults.

### 2.2. Participants

The target population consisted of 25 s and third-year students enrolled in the Nursing Degree at the University of Murcia (UM), Spain, during the 2022–2023 academic year; they voluntarily participated in the training program, which was not integrated into the Bachelor of Nursing curriculum.

A non-probabilistic sampling method was used, as the HFCS-based training was offered to all second and third-year students who wanted to participate in a volunteer program to mitigate loneliness in older adults.

The inclusion criteria were as follows: (1) students enrolled in the 2nd and 3rd year of the Nursing Degree, (2) took part in the volunteer training program to mitigate loneliness in older adults, and (3) signed the informed consent form. The exclusion criteria were as follows: (1) students who had medical or health conditions that could affect their active participation in the training program or the volunteer service.

### 2.3. Training Program

A training program was designed in two online modalities lasting 25 h and distributed over 3 differentiated weeks.

#### 2.3.1. Asynchronous Theoretical Training

Training took place using the virtual platform of the University of Murcia within the Sakai environment, which is a robust system that provides support to 4 million users in education environments to promote cooperative teaching, learning, and research [33]. The asynchronous volunteer training program included 5 modules (Table 1).

Each student accessed each of the modules in Sakai and viewed their contents. They had to pass a test on the contents before being able to access the next module.

In the asynchronous training, a calendar was proposed to ease completion. All participants had to complete the asynchronous online portion and pass an exam before taking part in the synchronous practical training.

#### 2.3.2. High-Fidelity Clinical Simulation-Based Synchronous Practical Training

The synchronous practical training took place using the online communication tool Zoom during an eminently practical session that lasted 5 h. Practical training was based on potential scenarios that students could face during the volunteer program HELPeN. In the volunteering program, the students must make a call once a week for a total of 36 weeks. A script was developed to help students during interventions and was implemented in each of the calls to older adults in order to favor effective interventions (Supplementary Materials S1).

Simulation Design Process:

The design of the simulation program was performed according to the guidelines from the International Nursing Association of Clinical and Simulation Learning (INACSL) [34].

Five clinical scenarios for telephone interviews were designed, which addressed different situations related to the telecare offered by the volunteers to older adults who suffered from social isolation and loneliness in a community context (Table 2). All scenarios were designed following international recommendations [34]. All standardized patients were selected and trained for the representation of roles to guarantee the fidelity of the scenarios. In phone call simulations, the students were given the script to favor the development of simulated calls (Supplementary Materials S1).

Each session was structured and comprised prebriefing, briefing, simulation, and structured debriefing according to the INACSL guidelines [35].

The first half-hour of the session was used for prebriefing and to establish a psychologically safe environment. For this, various group dynamics were implemented based on the practices proposed by Rudolph, Raemer, and Simon [36] and the INACSL’s good practices standards [37]. These were as follows: (a) detailed information about the development of the session, (b) clarify the expectations regarding the procedure of the online simulation session, and (c) information about the logistic details of the platform and the tools utilized in the online session. During clinical scenarios, the standardized patient turned the camera off to simulate a telephone call; (d) students took advantage of errors as opportunities for learning; and (e) a “fictional contract” with participants and an agreement of confidentiality and commitment with respect to other participants were used.

Once the prebriefing ended, simulated telephone interview scenarios were conducted. A briefing session took place before each simulated scenario, in which information on the proposed scenario was presented to participants. Afterwards, a simulated telephone interview took place, during which the facilitators turned the camera off and adopted the role of a simulated patient, to simulate a telephone call in which the student played the role of a volunteer who had to make different types of clinical and social decisions and manage resources via the Electronic and Digital History Record ICEBE platform, which was developed for the management of the HELPeN program. The observing students completed a checklist about the situation, with respect to the positive aspects and aspects that could be improved upon, to assist in posterior analyses and reflection on the scenario. When each simulated telephone call ended, a structured debriefing took place [38] in which possible real situations that could be experienced during the volunteering phase were discussed. The INACSL indications were followed in the debriefing session [39]. Each student actively engaged in simulation sessions, partaking in both the simulated case and the subsequent collaborative debriefing.

### 2.4. Data Collection Instrument

Socio-demographic (age and gender) and academic year characteristics were assessed.

To assess the participant’s knowledge, their knowledge relative to the asynchronous part of the course was measured objectively. This was performed by five professors who were members of the research team. They were experts in training and research in geriatric nursing, with more than five years of experience. The objective test comprised 24 multiple-choice questions with four options, of which one was correct. The questions were theoretical in nature and related to the contents of the course and volunteer program. This test was given at the start and end of the training program.

To assess the attitudes of the students towards older adults, Kogan’s attitudes towards old people (KAOP) scale was utilized [40]. This scale consists of 34 items with a Likert-type response scale of 6 points (from 1 = “strongly disagree” to 6 = “strongly agree”). Seventeen of these items were positively worded, while the remaining seventeen were negatively worded and had to be inverted. The total scores ranged from 34 to 204 points. The highest scores showed a positive attitude towards older adults, and a score that is equal to or higher than 102 indicated a positive attitude. The Spanish version of this scale [41] obtained a reliability scale similar to the original version (Cronbach value = 0.82), and it has an internal structure comprising two factors, coinciding with the items that were positively and negatively worded. Other instrument validation studies in Spain have later provided similar results [42]. This test was given at the start and the end of the training program.

To assess the satisfaction with the training program of the participants, the adapted version of the Students’ Satisfaction Scale was utilized [43]. Eighteen of the items were adapted and distributed into three dimensions: satisfaction, utility, and usability. The Likert-type response scale comprised 5 response options ranging from “strongly disagree” to “strongly agree”. After its application, a good internal consistency was obtained in the three dimensions (Cronbach value between 0.79 and 0.86), a value similar to that obtained in the original scale (Cronbach value between 0.78 and 0.93). The values of the item–total correlation ranged between 0.31 and 0.96, a value similar to that obtained in the original scale (0.31 and 0.94). This scale was given at the end of the training program.

Lastly, to assess the perceptions of the best educational practices via simulations, the Educational Practices Questionnaire (EPQ) was utilized [44]. El EPQ comprises 16 items that are grouped into four dimensions (active learning, collaboration, different ways of learning, and expectations). Each item is assessed using a Likert-type scale with 5 response options (1 = strongly disagree, 2 = disagree, 3 = undecided, 4 = agree, and 5 = strongly agree). The sum of the scores of all items indicates a greater recognition of the best educational practices in simulations. The Spanish version, adapted by Farrés-Tarafa et al. [45], showed that the EPQ obtained adequate psychometric properties in terms of the internal consistency and validity of the construct. The internal consistency was adequate for the questionnaire as a whole and for each of the dimensions (α ≥ 0.70). Confirmatory factor analysis showed an adequate fit of the structure of 4 factors, which is consistent with the original version [44]. This questionnaire was provided at the end of the training program.

### 2.5. Data Collection

A data collection form was designed using the measurement instruments described above, and the Google Forms online tool was used.

At first, the form with all measurement instruments (except for the Students’ Satisfaction Scale and the Educational Practices Questionnaire) was sent through the virtual classroom so that all participants could complete it and send it before the start of the training program.

Then, after the end of the synchronous part of the training program (clinical simulation), the data collection form with the measurement instruments was sent again.

### 2.6. Statistical Analysis

The data were analyzed with IBM SPSS Statistics software version 22.0 for Windows (IBM Corp., Armonk, NY, USA). A descriptive analysis was conducted relative to the study’s variables. The mean and standard deviation were utilized for quantitative variables, and frequencies and percentages were used for categorical ones.

As most measurement results did not obtain a normal distribution, the pre- and post-data were subjected to a Bootstrap analysis [46]. The comparison between the pre- and post-intervention scores was performed using Student’s *t*-test with a Bonferroni correction for multiple comparisons. For each variable, the effect size was calculated using Cohen’s d to assess the magnitude of the effect of the intervention, with the use of the values proposed by Ferguson [47], in which 0.41 indicated a small effect, 1.15 indicated a medium effect, and 2.70 indicated a large effect.

### 2.7. Ethical Considerations

The study, approved by the Ethics Committee of the University of Murcia, Spain (Approval Number: 3267/2021), involved students who willingly participated and had provided written informed consent after a prior explanation of the study’s objectives. The research was carried out in strict adherence to the principles and guidelines outlined in the Declaration of Helsinki [48].

## 3. Results

### 3.1. Sociodemographic and Professional Characteristics

The final sample comprised 25 nursing students who had a mean age of 27.44 (SD = 12.38) years old, with 76% (*n* = 19) being women. With respect to their academic year, 68% were enrolled in their 2nd year, and 32% in their 3rd year of the Nursing Degree.

### 3.2. Effect of the Training Program

After the analysis of the items from the variable “attitudes of the students towards older adults”, it became evident that the average overall scores achieved by the participants for each element increased following training. These differences were statistically significant across all elements, demonstrating a medium effect size (Table S1).

**Figure 1:**
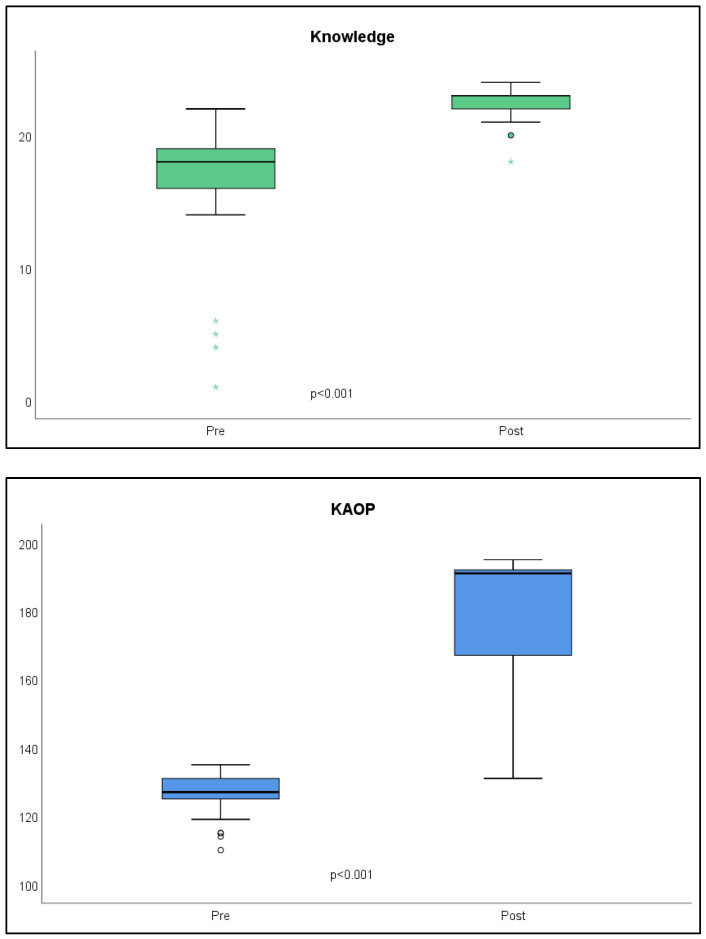
Scores obtained in the knowledge and attitudes towards older people variables before and after the training program. KAOP = Kogan’s attitudes towards older people. Knowledge: pre-M = 15.96; post-M = 22.36. KAOP: pre-M = 126.28; post-M = 175.52.

Figure 1 shows the total scores of variables “knowledge” and “attitudes of the students towards older people”. Statistically significant improvements were observed in the scores, with medium effect sizes, after the training program; the difference in means in the variable “knowledge” was 6.40 (t = 5.52, *p* < 0.001, d = 1.10), and in the variable “attitudes of the students towards older people”, it was 49.24 (t = 9.33, *p* < 0.001, d = 1.87).

### 3.3. Satisfaction and Perceptions of the Best Educational Practices in Simulation

**Figure 2:**
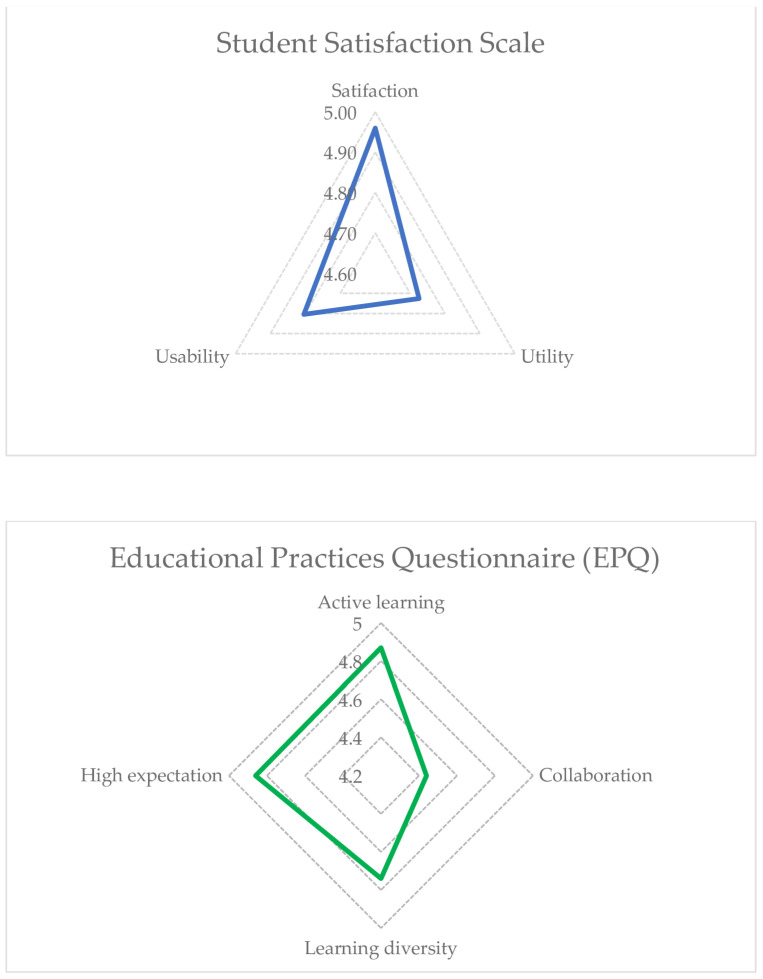
Scores obtained in the Student Satisfaction Scale and the Educational Practices Questionnaire.

The descriptive analysis of the Student Satisfaction Scale is shown in Table 3. As shown, all items obtained a mean score higher than 4.5 over 5, except for item 12 (M = 4.4, SD = 0.99), which indicates the participants’ high satisfaction. To ease the comprehension of the results, the percentage of the results is shown with the response scale. In all items, all scores obtained in the “agree/strongly agree” scale were 100%, except for items three “With the online modules I have learned more in this course than in a face-to-face course.” and twelve “The amount of work required for the modules of the HELPeN training program is adequate for understanding their content”. However, in these two items, agree/strongly agree responses comprised 96%. On the other hand, Figure 2 shows the itemized scores according to the response scale in the dimensions, in which it is observed that the participants obtained a score of 4.7 in all dimensions.

Table 4 shows the descriptive statistics data of the Educational Practices Questionnaire. As shown, all items obtained a mean score higher than 4.5 over 5, and 100%, scores were obtained relative to the “agree/strongly agree” response scale, except for item 12 “During the simulation, my peers and I had to work on the clinical situation together”. These scores indicate that the participants perceived that the high-fidelity clinical simulation session complied with the best educational practices in simulation standards. Figure 2 shows the weighted scores according to the response scale of the dimensions, where it is observed that the participants obtained scores higher than 4.5 in all dimensions except for the collaboration dimension (M = 4.44).

## 4. Discussion

We present a training program based on online clinical simulations to provide training to nursing students in order to reduce the social isolation and loneliness of older adults. Our results show the positive effects of this training program on the improvement in the knowledge of students, the positive attitudes towards older adults, the high satisfaction, and the positive perception of clinical-simulation-based practices. After the high-fidelity simulation training, our students improved their total scores with respect to all variables.

Our objective was to train nursing students to make simulated phone calls in order to palliate the social isolation and loneliness of older adults, and these phone calls met the requirements proposed by the INACSL [34] during their execution. Due to the online nature of the training, certain adjustments were made, including the absence of a specialized simulation room for the scenario. This experience was coherent, and it was adapted to the reality of other volunteering programs that address the social isolation and loneliness of older adults where telephone call training was not commonly provided, although these were highly used during the COVID-19 pandemic [49,50,51,52,53,54].

Although previous studies have shown the effectiveness of simulation-based training on geriatrics education [27], which improved attitudes towards older adults [28,29,55], there is a lack of studies that analyze its efficacy on the adequate management of nursing related to the social isolation and loneliness of older adults. Our simulation scenarios were centered on nursing care provided by students via telephone interviews, with diverse situations based on the telecare that volunteers must provide for the social isolation and loneliness of older adults; a script that could help in the development of the simulated call (Supplementary Materials S1) is provided. This differs from most of the studies up to the present, which addressed nursing only relative to specific geriatric syndromes, particularly dementia [56,57] or Alzheimer’s disease [58]. It must be underlined that the results from our study are similar to other studies that improved the attitudes towards older adults via simulation-based training [28,29].

Standardized patients were included in the simulated scenarios. Their inclusion was shown to be effective in other studies that utilized the same education strategy [27], which facilitated the experiential learning of students. Thus, our study indicates that the development of positive attitudes towards older adults improves when students have the opportunity to interact with older adults, which helps them understand what they feel and experience.

Regarding the faithfulness of the simulated scenarios, students expressed that the simulation was indeed realistic. This realism stemmed from the contextualization of the scenarios to closely resemble the actual volunteering situations that are likely encountered, thereby facilitating their application in clinical practice. The attainment of such a high level of fidelity in these simulated scenarios can be attributed to the adherence to established standards in both the design of simulation-based training and scenario development [34]. To achieve this goal, the instructors deactivated their cameras to emulate a real telephone call environment, enabling the students to concentrate solely on the audio aspect when conducting the interview. Additionally, the standardized patient received thorough training for each clinical scenario. This detail is of significant importance as it contributes to heightened fidelity, which in turn enhances the educational outcomes of the clinical simulation-based activity [59].

The high degree of satisfaction of the students and their perception that the high-fidelity clinical simulation-based session met the best educational practices in terms of simulation standards in the training program were perfectly in tune, with a very positive assessment provided by the students. These results are congruent with other studies that utilized face-to-face [60,61] and online [62,63] clinical simulation methodologies.

The results of the satisfaction with respect to the online learning modules showed that all items were positively scored by the participants, with mean high scores of 4.70 over 5 obtained in all dimensions (satisfaction, utility, and usability). Also, the scores obtained in the “agree/strongly agree” response scale were 100% relative to all items, except for items three “With the online modules I have learned more in this course than in a face-to-face course” and twelve “The amount of work required for the modules of the HELPeN training program is adequate for understanding their content”. This could be due to students’ preference for face-to-face learning and that the work overload perceived by students in the training program was high because the training course simultaneously took place with their official university courses.

Likewise, the results from the Educational Practices Questionnaire showed that the perception of the high-fidelity clinical simulation session met the best educational practices in terms of simulation standards and obtained high scores of 4.5 over 5 in all dimensions, except for the collaboration dimension. The collaboration weakness outlined in Figure 2 is to be expected given that the type of training was related to a single student talking with a “patient”, limiting collaborative elements within the training program. However, in our study, the utilization of collaborative debriefing sessions among participating students yielded notable benefits. These sessions facilitated a dynamic exchange of insights and perspectives, fostering a richer understanding of the challenges and strategies for addressing social isolation among aged adults.

These results are supported by the fact that the training program followed international recommendations on the design of activities based on clinical simulations [34], the use of a psychologically safe environment [36,64], and a structured debriefing [38,39].

Finally, it is worth emphasizing that the primary benefit of simulation-based training resides in its capacity to bridge the gap between theory and practical application, fostering a valuable learning experience from errors. Students underscored these aspects in their satisfaction survey, aligning with their recognition of effective educational practices within the realm of simulation, a consensus that is also supported by findings from prior research studies [65,66,67].

### Limitations

The present study is not without limitations. One methodological limitation pertained to the use of convenience sampling and the relatively modest sample size, thus constraining the generalizability of the findings. Nonetheless, to enhance the study’s external validity, the pre- and post-data underwent bootstrap analysis via a simulation involving 1000 participants. Another limitation, which was not very frequent, was the existence of technical problems during online simulation sessions (videoconference). However, these problems could be common in real phone calls given the lack of coverage or skills of older adults with respect to the use of a mobile phone, which would make the scenario more realistic. Another limitation of our study was the bias due to the use of self-reporting scales. However, all these instruments were validated, obtained high validity and reliability, and have been utilized in many international studies. Lastly, in future studies, long-term monitoring should be implemented to ensure that the knowledge and attitudes towards older adults of nursing students are maintained in the long term.

## 5. Conclusions

The online clinical simulation-based training initiative, emulating realistic scenarios via simulated telephone interactions, effectively addresses the educational requirements of nursing students engaging in a telecare volunteering program aimed at alleviating social isolation and loneliness among elderly individuals. In this sense, the study shows how training using a virtual modality via high-fidelity simulations is plausible, well received by students, and exhibits a high degree of learning consolidation.

The online clinical-simulation-based training program, characterized by its high-fidelity approach, enabled nursing students to enhance both their favorable attitudes towards older adults and their knowledge of addressing social isolation and loneliness in this demographic. Prior to encountering real-world scenarios, the cultivation of these competencies ideally takes place via simulated interactions with elderly individuals, affording students the chance to empathize with these individuals and comprehend their emotions and experiences. Also, the development of these skills can provide benefits that are directly related to performing high-quality telecare to palliate the social isolation and loneliness of older adults.

The online high-fidelity simulation was highly satisfactory and met the best educational practices in simulation, which defines it as a useful option in contexts in which the face-to-face training activities of students and health professionals are not possible, or it could be used to favor accessibility and aspects, such as professional and personal reconciliation.

Although our findings are promising, it is important to emphasise the need for further research aimed at implementing similar initiatives in actual practice. To effectively bridge the gap between education and practice, future studies should explore the sustained influence of simulation training on nursing students in order to provide high-quality care to older adults experiencing social isolation. Such research should also explore long-term benefits.

## Figures and Tables

**Table 1:** Asynchronous training modules.

MODULE	TITLE	HOURS
Module 1	Changes during Aging/Loneliness	2
Module 2	Communication techniques	3
Module 3	Assessment according to patterns of older adults	3
Module 4	Digital Electronic History Records ICEBE	2
Module 5	Reminiscence therapy	3

**Table 2:** Clinical scenarios.

Scenario	Learning Objectives
You call Juana, who is 70 years old, a widow for 2 years, without children. It is the first call session, she understands, but is suspicious; when we ask her, she answers everything with a Yes or No. When her husband was alive, they traveled and went to the retirement home, but since she became a widow, she only leaves the house to go to the health center to check her blood pressure. Finally, Juana participates in the call and collaborates.	- Apply call script. - Establish bond/circle of trust. - Redirect the call. - Plan the next call. - Presentation of the program and the student. - Record in call platform.
You call Irene, who is 73 years old, married and with 4 children who do not live with her. She was was a biology professor, and since she retired, she is taking care of her husband, who depends on her for all the basic activies of daily living (BADL) after suffering a stroke 8 years ago. Irene has hearing problems, so that communication is more difficult and needs the questions to be repeated multiple times. She tells us that she has always taken care of her husband, but needs professional help because she is very tired. Before, she used to go on walks, but now, given her situation, does not go out, not even to church, and does not meet her friends, although she is independent for all the BADL.	- Apply call script. - Maintain the bond. - Assess the state of health by patterns. - Identify pattern alteration. - Plan next call. - Record in call platform. - Record in evaluation platform. - Evaluate Pattern 1: Perception of health management.
In session 8, you call David, who is 74 years old, single, used to live with her sister, who passed away earlier this week. Does not stop crying, we cannot talk or follow the call script. He says that he cannot think about life without his sister, they have been together since they were children, and does not want to continue living, and he does not feel like talking today.	- Apply call script. - Maintain the bond. - Redirect the call. - Plan next call. - Record in call platform. - Record in notes platform.
You call Antonia in session 10, who is 73 years old, a widow for 30 years, and who lives with her 2 daughters and 3 grandchildren at home. Her daughters work in the hotel business and she is in charge of taking the grandchildren to school and taking care of them. In the previous session we asked her to write about the most relevant part of the topic discussed (her wedding), she tells us that she has not been able to do it because since she found the wedding photo she feels very sad. She remembers when her eldest daughter was born and how happy they were at that moment.	- Apply call script. - Maintain the bond. - Redirect the call. - Use reminiscence therapy. - Comment on the expression of feelings.
In session 18, you call Veronica, who is 66 years old, and divorced for 16 years; her daughter and her teenage granddaughter live at home. She is very angry because of a family argument, she tells us what has happened, the reason for the argument and other previous arguments, but we redirect the call. In the nursing assessment, she told us that she sometimes forgot to take the stomach protector and that she has never been vaccinated against the flu, because she has a phobia of needles. Every day she goes out for a walk alone at 07:00 am and she no longer leaves the house, her daughter and her granddaughter do the shopping.	- Apply call script. - Maintain the bond. - Redirect the call. - Maintain the bond. - Education for health: medication. - Education for health: vaccines. - Proposal for change to go for a walk accompanied and visit an emblematic place. - Plan next call. - Record in call platform. - Record in notes platform.

**Table 3:** Descriptive statistics of the items and dimensions of the adapted version of the Student Satisfaction Scale.

		1	2	3	4	5
Items	M (SD)	*n* (%)				
Dimension 1. Satisfaction						
I am satisfied with the modules completed in the HELPeN training program.	4.96 (0.35)	-	-	-	1 (4)	24 (96)
2. I would recommend other people to do the HELPeN training program.	4.96 (0.44)	-	-	-	1 (4)	24 (96)
Dimension 2. Utility						
3. With the online modules I have learned more in this course than in a face-to-face course.	4.68 (0.91)	-	1 (4)	-	5 (20)	19 (76)
4. The online course of the HELPeN training program encouraged me to rethink my understanding of some aspects of the subject	4.84 (0.74)	-	-	-	4 (16)	21 (84)
5. The modules of the HELPeN training program used in this course facilitated my learning.	4.76 (0.6)	-	-	-	6 (24)	19 (74)
6. The preparation/completion of the final tests in each module facilitated my learning.	4.52 (0.83)	-	-	-	12 (48)	13 (52)
7. The materials and documents helped me direct my studying and learning	4.68 (0.79)	-	-	-	8 (32)	17 (68)
8. The HELPeN training program helped me to be prepared to volunteer.	4.68 (0.9)	-	-	-	8 (32)	17 (68)
9. I used the HELPeN training program to learn the content before carrying out the intervention with the elderly.	4.92 (0.45)	-	-	-	2 (8)	23 (92)
Dimension 3. Usability						
10. The modules of the HELPeN training program clarified the expectations of what it is necessary to understand to obtain a good result in the telephone intervention with the elderly.	4.84 (0.64)	-	-	-	4 (16)	21 (84)
11. From the beginning, the HELPeN training program made it clear to me what I had to learn in each unit.	4.96 (0.4)	-	-	-	1 (4)	24 (96)
12. The amount of work required for the modules of the HELPeN training program is adequate for understanding their content.	4.4 (0.99)	-	1 (4)	-	12 (48)	12 (48)
13. The HELPeN training program gave me easy access to online/digital learning resources.	4.8 (0.57)	-	-	-	5 (20)	20 (80)
14. The HELPeN training program allowed me to be responsible for my own learning.	4.8 (0.65)	-	-	-	5 (20)	20 (80)
15. The HELPeN training program helped me focus my attention on specific areas that I needed.	4.88 (0.69)	-	-	-	3 (12)	22 (88)
16. The management of the platform and access to the resources offered by the HELPeN training program are simple	4.8 (0.41)	-	-	-	5 (20)	20 (80)
17. The viewing of the multimedia material is was not difficult.	4.96 (0.59)	-	-	-	1 (4)	24 (96)
18. Connect helped me to focus my attention on specific areas of need.	4.8 (0.53)	-	-	-	5 (20)	20 (80)

**Table 4:** Descriptive statistics of the items and dimensions of the Educational Practices Questionnaire.

		1	2	3	4	5
Items	M (SD)	*n* (%)				
Dimension 1. Active learning						
I had the opportunity during the simulation activity to discuss the ideas and concepts taught in the course with the teacher and other students.	4.92 (0.28)	-	-	-	2 (8)	23 (92)
2. I actively participated in the debriefing session after the simulation.	4.72 (0.46)	-	-	-	(7 (28)	18 (72)
3. I had the opportunity to put more thought into my comments during the debriefing session.	4.84 (0.37)	-	-	-	4 (16)	21 (84)
4. There were enough opportunities in the simulation to find out if I clearly understand the material.	4.80 (0.41)	-	-	-	5 (20)	20 (80)
5. I learned from the comments made by the teacher before, during, or after the simulation.	4.92 (0.28)	-	-	-	2 (8)	23 (92)
6. I received cues during the simulation in a timely manner.	4.80 (0.41)	-	-	-	5 (20)	20 (80)
7. I had the chance to discuss the simulation objectives with my teacher.	4.92 (0.28)	-	-	-	2 (8)	23 (92)
8. I had the opportunity to discuss ideas and concepts taught in the simulation with my instructor.	4.96 (0.2)	-	-	-	1 (4)	24 (96)
9. The instructor was able to respond to the individual needs of learners during the simulation.	4.92 (0.28)	-	-	-	2 (8)	23 (92)
10. Using simulation activities made my learning time more productive.	4.92 (0.28)	-	-	-	2 (8)	23 (92)
Dimension 2. Collaboration						
11. I had the chance to work with my peers during the simulation.	4.56 (0.51)	-	-	-	11 (44)	14 (56)
12. During the simulation, my peers and I had to work on the clinical situation together.	4.32 (0.63)	-	-	2 (8)	13 (52)	10 (40)
Dimension 3. Learning diversity						
13. The simulation offered a variety of ways in which to learn the material.	4.72 (0.46)	-	-	-	7 (28)	18 (72)
14. This simulation offered a variety ways of assessing my learning.	4.76 (0.44)	-	-	-	6 (24)	19 (76)
Dimension 4. Hight expectation						
15. The objectives for the simulation experience were clear and easy to understand.	4.88 (0.33)	-	-	-	3 (12)	22 (88)
16. My instructor communicated the goals and expectations to accomplish during the simulation.	4.84 (0.37)	-	-	-	4 (16)	21 (84)

## Data Availability

The data are available upon email request to the corresponding authors.

## Supplementary Materials

The following supporting information can be downloaded at https://www.mdpi.com/article/10.3390/healthcare11182587/s1. Call script and objectives of the telephone interventions in the sessions with the elderly. Table S1: Scores obtained in the items from the KAOP questionnaire pre- and post-training.
