# Supplementary material for: Effects of a Clinical Simulation-Based Training Program for Nursing Students to Address Social Isolation and Loneliness in the Elderly: A Quasi-Experimental Study
Source: Healthcare (Basel). 2023 Sep 19;11(18):2587. doi: 10.3390/healthcare11182587 (PMC10531334; doi:10.3390/healthcare11182587)
Supplement: Supplementary file 1 [file healthcare-11-02587-s001.zip › Supplementary Materials S1.pdf]

Cátedra Poncemar de Gerontología

UNIVERSIDAD DE MURCIA

PROYECTO HELPeN

# CALL SCRIPT AND OBJECTIVES OF THE TELEPHONE INTERVENTIONS IN THE SESSIONS WITH THE ELDERLY

**TELEPHONE INTERVENTIONS: OBJECTIVES AND SCRIPT ACCORDING TO SESSION.**

## TELEPHONE INTERVENTONS WITH THE ELDERLY: OBJECTIVES OF THE SESSIONS AND SCRIPTS FOR EACH CALL

1 weekly phone call and 36 sessions.

Sessions 1, 2 and 3: Establish the voluntary-elderly link.

Sessions 4, 5, 6 and 7: Health Counseling, assessment patterns 1-4.

Sessions 8, 9 and 10: Accompaniment and Reminiscence Therapy.

Sessions 11, 12 and 13. Health Counseling, assessment patterns 5-11.

Sessions 14, 15, 16 and 17: Accompaniment and Reminiscence Therapy.

Sessions 18, 19 and 20: Guidance towards change

Sessions 21 and 22: Accompaniment and Reminiscence Therapy.

Sessions 23 and 24: Guidance towards change

Sessions 25 and 26: Accompaniment and Reminiscence Therapy.

Sessions 27, 28 and 29: Guidance towards change

Sessions 30 and 31: Accompaniment and Reminiscence Therapy.

Sessions 32 and 33: Guidance towards change

Sessions 34 and 35: Accompaniment and Reminiscence Therapy.

Session 36: Evaluation sessions.

**<u>TELEPHONE INTERVENTIONS: OBJECTIVES AND SCRIPT ACCORDING TO SESSION.</u>**

**SESSION 1:** First telephone contact of the volunteer with the elderly and establishment of the link.

**Objectives:**

- Establish first contact
- Establish bond/circle of trust
- Specify the time and date of calls.
- Presentation of the program
- Presentation of the student
- Discover the likes/hobbies and social relationships

**SCRIPT SESSION 1:**

Good afternoon XXX, my name is XXX, I am a nursing student and as your nurse commented, we called you to follow up and accompany you.

Tell me, how are you physically?

How are you emotionally?

Who do you live with at home?

Do you have any hobbies that you currently maintain?

Do you like to go to a community site (association, petanque club, housewives club)?

**TELEPHONE INTERVENTIONS: OBJECTIVES AND SCRIPT ACCORDING TO SESSION.**

I don't know if this has been explained to you, but the program consists of calling you weekly and talking for about 30 minutes, for a total of 36 sessions or calls. The objective of the calls is to provide company, listen to you and improve your quality of life. Would you like me to explain anything else, or do you have any questions?

Would it be convenient for you if I call you next week at the same time as today?

If it is not convenient for you, what day of the week is best for you and at what time?

If the day or time does not suit you in any week, no problem, we could change it, since I will remind you of the next call every week.

Specify the day and time of the telephone session for the following week.

Record the most important aspects of the session in the field journal.

**Observations:**

**TELEPHONE INTERVENTIONS: OBJECTIVES AND SCRIPT ACCORDING TO SESSION.**

**SESSION 2:** Continuity in the establishment of the bond.

**Objectives:**

- Encourage contact
- Establish bond/circle of trust
- Specify the time and date of calls.
- Remind them about the program
- Discover the likes/hobbies and social relationships
- Discover aspects that worry or worry you

**SCRIPT SESSION 2:**

Good afternoon, XXXX, this is XXXX. Do you remember that we were talking last week about your hobbies, who you live with, and I explained the program to you, and we scheduled the call for this week, which aims to provide company, listen to you, and improve your quality of life?

Do you want to ask me something that was not clear to you last week?

Tell me, how are you this week? Has there been any change in your physical health from the previous week?

And in your state of mind with respect to the past week?

Have you done any different activities or hobbies, or have you gone to the association this week?

**TELEPHONE INTERVENTIONS: OBJECTIVES AND SCRIPT ACCORDING TO SESSION.**

And tell me, do you receive visitors? Do your family or friends come to see you?

Do you have a concerns that have come up this week or something that has happened to you that I can help you with?

Well, next week I will call you again... (Specify the day and time of the telephone session for the following week).

Record the most important aspects of the session in the field journal.

**Observations:**

**TELEPHONE INTERVENTIONS: OBJECTIVES AND SCRIPT ACCORDING TO SESSION.**

**SESSION 3:** Strengthen the bond.

**Objectives:**

- Encourage contact
- Strengthen the bond/circle of trust
- Specify the time and date of calls.
- Remind them about the program
- Talk about likes/hobbies and social relationships
- Discover aspects that worry or disturb them
- Evaluate the satisfaction with the call

**SCRIPT SESSION 3:**

Good afternoon, XXXX, this is XXXX. Do you remember that last week we were talking about your hobbies, who you live with, and I explained the program to you and we scheduled the call for this week, which aims to provide you with company, listen to you, and improve your quality of life?

Do you want to ask me something that was not clear to you last week?

Tell me, how are you doing this week?

Has there been any change in your physical health from the previous week?

And in your state of mind with respect to the past week?

Have you done any different activities or hobbies, or have you gone to the association this week?

**TELEPHONE INTERVENTIONS: OBJECTIVES AND SCRIPT ACCORDING TO SESSION.**

Have you received visitors in the last week? (If they told us YES to visits on the last call)

How do you feel about the calls?

Would you like us to change something?

Do you have a concern that has come up this week or something that has happened to you that I can help you with?

Well, next week I will call you again. (Specify the day and time of the telephone session for the following week.)

Record the most important aspects of the session in the field journal.

**Observations:**

## TELEPHONE INTERVENTIONS: OBJECTIVES AND SCRIPT ACCORDING TO SESSION.

**SESSION 4:** Start with health advice. Pattern 1: Perception of health management.

**Objectives:**

- Keep the bond strong
- Assess the state of health
- Assess toxic habits
- Identify pattern alteration
- Assess adequate consumption of medication.
- Assess vaccination
- Assess allergies
- Specify the time and date of calls.

**SCRIPT SESSION 4:**

Good afternoon XXXX, this is XXXX

How are you? How has the week been? Any change or situation that has happened to you?

When you were young, did you ever smoke or drink?

Is there any medication or food that makes you feel bad? (allergies)

Now with the whole issue of the pandemic, that vaccinations are very important now, do you usually get vaccinated for the flu, pneumonia and so on?

Tell me, do you sometimes forget to take your medication or do you have trouble remembering it? What do you do so you don't forget? (Who picks it up, if they or a family

member prepares it, if they have a pill box or sets an alarm to take it, if they has a routine...)

Well, next week I will call you again... (Specify the day and time of the telephone session for the following week.)

Record the most important aspects of the session in the field journal. .

**Observations:**

**TELEPHONE INTERVENTIONS: OBJECTIVES AND SCRIPT ACCORDING TO SESSION.**

**SESSION 5:** Health advice. Pattern 2: Nutritional, metabolic.

**Objectives:**

- Keep the bond strong
- Assess nutrition
- Assess fluid intake
- Identify pattern alteration
- Assess skin changes
- Specify the time and date of calls.

**SCRIPT SESSION 5:**

Good afternoon XXXX, this is XXXX

How are you? How has the week been?

Any change or situation that has happened to you?

Do you eat everything? (food groups, what they do not like, things do not suit them, if they have any problems eating (teething, nausea, loss of appetite, dyspepsia) take some kind of supplement, follow some kind of diet, if they eat at home or out, if they cook or not, )

Let's see, tell me, when you get up what do you drink? And at mid-morning, do you have a drink? Then at lunchtime? Do you usually have a snack? What do you have for dinner? And before going to bed, if you have had dinner early, do you have a drink? (Planning meals)

How much water do you drink a day? In glasses or in bottles how many? (Any other drink, coffee, milk, beer, wine…)

And could you tell me how much you weigh and how tall you are? (If they don't know and they have a scale, plan for them to weigh themselves and tell us the following week and if they don't have a scale and don't know it, mark it as non-assessable)

Have you noticed that you have gained or lost weight in the last month?

Do you have any wound or injury?

Do you clean it yourself or does the nurse do it?

Do you have a concern that has come up this week or something that has happened to you that I can help you with?

Well, next week I will call you again... (Specify the day and time of the telephone session for the following week.)

Record the most important aspects of the session in the field journal.

**Observations:**

**TELEPHONE INTERVENTIONS: OBJECTIVES AND SCRIPT ACCORDING TO SESSION.**

**SESSION 6:** Health advice. Pattern 3: Elimination.

**Objectives:**

- Keep the bond strong
- Assess for fecal incontinence
- Assess for urinary incontinence
- Identify pattern alteration
- Assess the type of help you use or if you would need help
- Assess another type of loss, amount of exudate or fluid.
- Specify the time and date of calls.

**SCRIPT SESSION 6:**

Good afternoon XXXX, this is XXXX

How are you? How has the week been? Any change or situation that has happened to you?

Do you go to the bathroom regularly? (No. of stools, stool type, color, any change, take some type of medicine to help, hemorrhoids, gas, pain, incontinence, ostomies)

Do you have a problem urinating? (Number of times, pain, nocturia, hematuria, retention, bladder catheter, suprapubic catheter, urostomies, nephrostomy,)

If the patient is incontinent, assess what type of aid is used. What kind of device do you use for incontinence? (Diaper by day, by night,)

**TELEPHONE INTERVENTIONS: OBJECTIVES AND SCRIPT ACCORDING TO SESSION.**

If necessary because in the previous call they mentioned having injuries... assess the loss due to exudate, drainage....

Well, next week I will call you again... (Specify the day and time of the telephone session for the following week.)

Record the most important aspects of the session in the field journal.

**Observations:**

**TELEPHONE INTERVENTIONS: OBJECTIVES AND SCRIPT ACCORDING TO SESSION.**

**SESSION 7:** Health advice. Pattern 4: exercise-activity.

**Objectives:**

- Keep the bond strong
- Use reminiscence therapy
- Comment on the expression of feelings
- Identify a significant experience
- Schedule the next call
- Specify the time and date of calls.

**SCRIPT SESSION 7:**

Good afternoon XXXX, this is XXXX

How are you? How has the week been? Any change or situation that has happened to you?

Do you do any type of exercise or activity?

Do you get tired or feel energetic?

Do you use any support device for walking? (Maintain balance, stability,)

Can you dress yourself? Wash and eat alone? Go to the toilet alone?

Do you like to dress up when you go out?

**TELEPHONE INTERVENTIONS: OBJECTIVES AND SCRIPT ACCORDING TO SESSION.**

Do you have any respiratory problems? Do you need any medication (inhalers, drugs)?

Do you use oxygen or a sleeping device? In case of a respiratory alteration, assess physiotherapy and secretions, breathing sounds and distress.

And could you tell me what your blood pressure and heart rate are? (If they don't know and they have a blood pressure monitor or the possibility of going to the pharmacy/health center, plan so that they give it to you the following week, and if not and they don't know it, mark it as not measurable)

Have you fallen lately?

Do you have a concern that has come up this week or something that has happened to you that I can help you with?

Well, next week I will call you again... (Specify the day and time of the telephone session for the following week.)

Record the most important aspects of the session in the field journal.

**Observations:**

**TELEPHONE INTERVENTIONS: OBJECTIVES AND SCRIPT ACCORDING TO SESSION.**

**SESSION 8:** Accompaniment and Reminiscence Therapy.

**Objectives:**

- Use reminiscence therapy
- Comment on the expression of feelings
- Identify a significant experience
- Schedule the next call
- Specify the time and date of calls.

**SCRIPT SESSION 8:**

Good afternoon XXXX, this is XXXX

How are you? How has the week been? Any change or situation that has happened to you?

In this session and in the following sessions we are going to work on memory, recollection and the feelings associated with those memories.

Could you identify moments, circumstances or experiences from your past that have been significant to you? Provide some time and wait for the elder to identify something. If the elder does not identify anything for you, you can give examples: when they got married, the birth of children, work, and experiences with friends, the children's school, anecdotes...

**TELEPHONE INTERVENTIONS: OBJECTIVES AND SCRIPT ACCORDING TO SESSION.**

With the elder, identify a theme for the next session (one of the themes that they have identified). On the next call, what topic would you like us to remember? (For the next session, remember during this week about the agreed topic and tell me what you want about the topic. (Narration of the story) and if possible, for next week, could you locate a photo related to the topic to be discussed? (Goal: stimulate memory)

Do you have any questions about what we have been talking about?

Well, next week I will call you again... (Specify the day and time of the telephone session for the following week.)

Record the most important aspects of the session in the field journal.

**Observations:**

**TELEPHONE INTERVENTIONS: OBJECTIVES AND SCRIPT ACCORDING TO SESSION.**

**SESSION 9:** Accompaniment and Reminiscence Therapy.

**Objectives:**

- Keep the bond strong
- Use reminiscence therapy
- Comment on the expression of feelings
- Facilitate the identification of positive aspects of the experience
- Facilitate the identification of negative aspects of the experience
- Facilitate the narration of the experience lived
- Discuss the expression of both positive and negative aspects of the experience.
- Schedule the next call
- Specify the time and date of calls.
- Identify a significant experience

**SCRIPT SESSION 9:**

Good afternoon XXXX, I am XXXX

How are you? How has the week been? Any change or situation that has happened to you?

Reminder of the previous session: remind them about the selected topic to remember. Have you found a photo or image that reminds you of the topic?

Tell me, how did it happen? Facilitate the expression of feelings and the narration of the event. Introduce open-ended questions as needed: When did it happen? What else do you remember? Who were you with?

How did you feel? (Encourage verbal expression of feelings, both positive and negative, about the past event)

**TELEPHONE INTERVENTIONS: OBJECTIVES AND SCRIPT ACCORDING TO SESSION.**

Keep the focus of the session mainly on the process rather than the end result. For example: How did you experience the situation, what positive aspects did you experience? And the negative aspects?

Provide support, encouragement and empathy: Incorporate, as needed, expressions such as: "I understand", "I understand what would happen"...

If necessary, use direct questions to refocus on the event. For example: going back to the event… could you describe how you felt? Or how did you deal with the situation?

Recognize the coping skills used in the event. For example: as you tell me... (use the words used by the older person to deal with the situation)

It would be positive if everything you are telling me during this week was written in a notebook. would this be possible? could do it?

Closing the session: Mr/Ms XXX, we have to end the session, we will continue next week.

With the elder, identify a theme for the next session (one of the themes that you have identified). On the next call, what topic would you like us to remember? (For the next session, remember about the agreed topic during the week and tell me what you want

about the topic. (Narration of the story) and if possible, for next week, could you locate a photo related to the topic to be discussed? (Goal: stimulate memory)

Do you have any questions about what we have been talking about?

Well, next week I will call you again... (Specify the day and time of the telephone session for the following week.)

Record the most important aspects of the session in the field journal.

**Observations:**

**TELEPHONE INTERVENTIONS: OBJECTIVES AND SCRIPT ACCORDING TO SESSION.**

**SESSION 10:** Accompaniment and Reminiscence Therapy.

**Objectives:**

- Keep the bond strong
- Use reminiscence therapy
- Comment on the expression of feelings
- Facilitate the identification of positive aspects of the experience
- Facilitate the identification of negative aspects of the experience
- Facilitate the narration of the lived experience
- Schedule the next call
- Specify the time and date of calls.
- Comment on the expression of both positive and negative aspects of the experience.

**SCRIPT SESSION 10:**

Good afternoon XXXX, this is XXXX

How are you? How has the week been? Any change or situation that has happened to you?

Reminder from the previous session: do you remember that I told you that it would be good if you wrote down the most relevant aspects about what you told me last week? Could you do it? Do you want us to talk about it?

The elders talk about what they have written (let them tell us)

**TELEPHONE INTERVENTIONS: OBJECTIVES AND SCRIPT ACCORDING TO SESSION.**

Remember the selected topic to remember. Have you found a photo or image that reminds you of the topic?

Tell me, how did it happen? Facilitate the expression of feelings and the narration of the event. Introduce open-ended questions as needed: When did it happen? What else do you remember? Who were you with?...

How did you feel? (Encourage verbal expression of feelings, both positive and negative, about the past event)

Keep the focus of the session mainly on the process rather than the end result. For example: How did you experience the situation, what positive aspects did you experience? And the negative aspects?

Provide support, encouragement and empathy: Incorporate, as needed, expressions such as: "I understand", "I understand what would happen"...

If necessary, use direct questions to refocus on the event. For example: going back to the event… could you describe how you felt? Or how did you deal with the situation?

Recognize the coping skills used in the event. For example: as you tell me... (Use the words used by the older person to deal with the situation)

**TELEPHONE INTERVENTIONS: OBJECTIVES AND SCRIPT ACCORDING TO SESSION.**

It would be positive if everything you are telling me during this week was written in a notebook. Would this be possible? Could do it?

Closing the session: Mr/Ms XXX, we have to end the session, we will continue next week.

Do you have any questions about what we have been talking about?

Well, next week I will call you again... (Specify the day and time of the telephone session for the following week.)

Record the most important aspects of the session in the field journal.

**Observations:**

**TELEPHONE INTERVENTIONS: OBJECTIVES AND SCRIPT ACCORDING TO SESSION.**

**SESSION 11:**

Health advice. Pattern 5: Sleep, Pattern 6: Cognitive-Perceptive and Pattern 9: Sexuality-Reproduction.

**Objectives:**

- Keep the bond strong
- Assess sleep disturbance
- Assess sleep aids
- Identify sleep pattern disturbance.
- Assess mood
- Assess vision and hearing impairment
- Assess the use of hearing and vision aids
- Pain assessment
- Identify alteration of the cognitive-perceptive pattern.
- Assess sexual alteration
- Assess reproductive function
- Identify alteration of the sexuality-reproduction pattern.
- Specify the time and date of calls.

**SCRIPT SESSION 11:**

Good afternoon XXXX, this is XXXX

How are you? How has the week been? Any change or situation that has happened to you?

Do you have any trouble sleeping, or have a hard time falling asleep?

Do you need pharmacological help to sleep? Do you sleep during the day? (How many hours do you sleep during the day and at night, changes)

**TELEPHONE INTERVENTIONS: OBJECTIVES AND SCRIPT ACCORDING TO SESSION.**

How are you feeling? Do you have trouble concentrating or remembering?

Do you have vision or hearing problems? If so, do you use help to fix it?

Does anything hurt? From 1 to 10? And do you use any kind of help (medication, postural hygiene) for pain?

When was your last checkup with the gynecologist or urologist? Do you have any problems? Have you had any problems with fertility? Are your sexual relations satisfactory?

Do you have a concern that has come up this week or something that has happened to you that I can help you with?

Well, next week I will call you again... (Specify the day and time of the telephone session for the following week.)

Record the most important aspects of the session in the field journal.

**Next week a colleague will call you to ask you some questions.**

**Observations:**

**TELEPHONE INTERVENTIONS: OBJECTIVES AND SCRIPT ACCORDING TO SESSION.**

**SESSION 12:** Health advice. Pattern 8: Role-Relationships, Pattern 11: Values – Beliefs.

**Objectives:**

- Keep the bond strong
- Assess changes in recent years
- Assess relationships
- Evaluate the activities
- Assess mistreatment or violence
- Assess the existence of a primary caretaker or dependents
- Assess religious practices
- Evaluate future expectations
- Identify pattern alteration
- Specify the time and date of calls.

**SCRIPT SESSION 12:**

Good afternoon XXXX, this is XXXX

How are you? How has the week been? Any change or situation that has happened to you?

Have you recently lost a family member/friend? How did you deal with it? How did you experience that moment? Did you feel lonely?

Who do you associate with most often? Do you feel lonely?

Are you caring for anyone or is someone caring for you? (If so, ask if the main caregiver is a family member, professional caregiver, caregiver, or live-in family? Or, is he or she a hired home help? Does the caregiver know their pathologies and treatment? )

**TELEPHONE INTERVENTIONS: OBJECTIVES AND SCRIPT ACCORDING TO SESSION.**

Is there someone close treating you in a manner that is not correct or that causes you fear or discomfort?

Do you regularly participate in religious practices?

Do you get the things you want? Would you like to get something?

Well, next week I will call you again... (Specify the day and time of the telephone session for the following week.)

Record the most important aspects of the session in the field journal.

**Observations:**

**TELEPHONE INTERVENTIONS: OBJECTIVES AND SCRIPT ACCORDING TO SESSION.**

**SESSION 13:** Health advice. Pattern 10: Adaptation-tolerance to stress.

**Objectives:**

- Keep the bond strong
- Evaluate lack / desire of information
- Assess the feeling of stress
- Assess changes in recent years
- Evaluate response to stressful situations
- Evaluate negative thoughts and attitude towards the disease
- Identify pattern alteration
- Specify the time and date of calls.

**SCRIPT SESSION 13:**

Good afternoon XXXX, this is XXXX

How are you? How has the week been? Any change or situation that has happened to you?

In recent years, have there been any major changes? How did you feel about that change? Have you felt fear or anxiety?

How do you normally feel, relaxed or stressed? Do you take any medication for your nerves?

When you have a problem, how do you deal with it? Who do you ask for help to talk about your things?

**TELEPHONE INTERVENTIONS: OBJECTIVES AND SCRIPT ACCORDING TO SESSION.**

Have you had negative thoughts about your situation? Have you had any attempts to harm yourself? What is your attitude towards your illness?

Well, next week I will call you again... (Specify the day and time of the telephone session for the following week.)

Record the most important aspects of the session in the field journal.

**Observations:**

**TELEPHONE INTERVENTIONS: OBJECTIVES AND SCRIPT ACCORDING TO SESSION.**

**SESSION 14:** Accompaniment and Reminiscence Therapy.

**Objectives:**

- Keep the bond strong
- Use reminiscence therapy
- Comment on the expression of feelings
- Identify a significant experience
- Schedule the next call
- Specify the time and date of calls.

**SCRIPT SESSION 14:**

Good afternoon XXXX, this is XXXX

How are you? How has the week been? Any change or situation that has happened to you?

Reminder of the previous session: remember the selected topic to remember. Remember that we were talking about significant experiences that were important to you. In these weeks, have you remembered any other important event? Or has a situation happened that reminded you of something else that was important?

In the last call we had to remember events, we were remembering XXXXXXXX, and we agreed that if you could, to write these memories, have you been able to do it? Would you like to talk about it? (If they have not done so, we agree on another situation to talk about in this session and if they have, we will talk about it)

**TELEPHONE INTERVENTIONS: OBJECTIVES AND SCRIPT ACCORDING TO SESSION.**

Tell me, how did it happen? Facilitate the expression of feelings and the narration of the event. Introduce open-ended questions as needed: When did it happen? What else do you remember? Who were you with?...

How did you feel? (Encourage verbal expression of feelings, both positive and negative, about the past event)

Keep the focus of the session mainly on the process rather than the end result. For example: How did you experience the situation, what positive aspects did you experience? And the negative aspects?

Provide support, encouragement and empathy: Incorporate, as needed, expressions such as: "I understand", "I understand what would happen"...

If necessary, use direct questions to refocus on the event. For example: going back to the event… could you describe how you felt? Or how did you deal with the situation?

Recognize the coping skills used in the event. For example: as you tell me... (Use the words used by the older person to deal with the situation)

It would be positive if everything you are telling me during this week was written in a notebook. Would this be possible? Could do it?

Closing the session: Mr/Ms XXX, we have to end the session, we will continue next week.

**TELEPHONE INTERVENTIONS: OBJECTIVES AND SCRIPT ACCORDING TO SESSION.**

With the elder, identify a theme for the next session (one of the themes that you have identified). On the next call, what topic would you like us to remember? (For the next session, remember about the agreed topic during the week and tell me what you want about the topic. (Narration of the story) and if possible, for next week, could you locate a photo related to the topic to be discussed? (Goal: stimulate memory)

Do you have any questions about what we have been talking about?

Well, next week I will call you again... (Specify the day and time of the telephone session for the following week.)

Record the most important aspects of the session in the field journal.

**Observations:**

## TELEPHONE INTERVENTIONS: OBJECTIVES AND SCRIPT ACCORDING TO SESSION.

**SESSION 15:** Accompaniment and Reminiscence Therapy.

**Objectives:**

- Keep the bond strong
- Use reminiscence therapy
- Comment on the expression of feelings
- Facilitate the identification of positive aspects of the experience
- Facilitate the identification of negative aspects of the experience
- Facilitate the narration of the lived experience
- Discuss the expression of both positive and negative aspects of the experience.
- Schedule the next call
- Specify the time and date of calls.
- Identify a significant experience

**SCRIPT SESSION 15:**

Good afternoon XXXX, this is XXXX

How are you? How has the week been? Any change or situation that has happened to you?

Reminder of the previous session: remember the selected topic to remember. Remember that we were talking about significant experiences that were important to you. In these weeks, have you remembered any other important event? Or has a situation happened that reminded you of something else that was important?

We agreed that if you could, to write these memories, have you been able to do it? Would you like to talk about it? (If they have not done so, we agree on another situation to talk

about in this session and if they have, we will talk about it). Propose talking about the birth of their children.

Tell me, how did it happen? Facilitate the expression of feelings and the narration of the event. Introduce open-ended questions as needed: When did it happen? What else do you remember? Who were you with?...

How did you feel? (Encourage verbal expression of feelings, both positive and negative, about the past event)

Keep the focus of the session mainly on the process rather than the end result. For example: How did you experience the situation, what positive aspects did you experience? And the negative aspects?

Provide support, encouragement and empathy: Incorporate, as needed, expressions such as: "I understand", "I understand what would happen"...

If necessary, use direct questions to refocus on the event. For example: going back to the event… could you describe how you felt? Or how did you deal with the situation?

Recognize the coping skills used in the event. For example: as you tell me... (Use the words used by the older person to deal with the situation)

**TELEPHONE INTERVENTIONS: OBJECTIVES AND SCRIPT ACCORDING TO SESSION.**

It would be positive if everything you are telling me during this week was written in a notebook. Would this be possible? Could do it?

Closing the session: Mr/Ms XXX, we have to end the session, we will continue next week.

With the elder, identify a theme for the next session (one of the themes that you have identified). On the next call, what topic would you like us to remember? (For the next session, remember about the agreed topic during the week and tell me what you want about the topic. (Narration of the story) and if possible, for next week, could you locate a photo related to the topic to be discussed? (Goal: stimulate memory)

Do you have any questions about what we have been talking about?

Well, next week I will call you again... (Specify the day and time of the telephone session for the following week.)

Record the most important aspects of the session in the field journal.

**Observations:**

**TELEPHONE INTERVENTIONS: OBJECTIVES AND SCRIPT ACCORDING TO SESSION.**

**SESSION 16:** Accompaniment and Reminiscence Therapy.

**Objectives:**

- Keep the bond strong
- Use reminiscence therapy
- Comment on the expression of feelings
- Facilitate the identification of positive aspects of the experience
- Facilitate the identification of negative aspects of the experience
- Facilitate the narration of the lived experience
- Discuss the expression of both positive and negative aspects of the experience.
- Schedule the next call
- Specify the time and date of calls.
- Identify a significant experience

**SCRIPT SESSION 16:**

Good afternoon XXXX, this is XXXX

How are you? How has the week been? Any change or situation that has happened to you?

Reminder of the previous session: remember the selected topic to remember. Did you find an object/photo that reminds you of the subject?

We agreed that if you could, to write these memories, have you been able to do it? Would you like to talk about it? (If they have not done so, we agree on another situation to talk about in this session and if they have, we will talk about it). Propose talking about their wedding or a child’s wedding or the death of a loved one.

**TELEPHONE INTERVENTIONS: OBJECTIVES AND SCRIPT ACCORDING TO SESSION.**

Tell me, how did it happen? Facilitate the expression of feelings and the narration of the event. Introduce open-ended questions as needed: When did it happen? What else do you remember? Who were you with?...

How did you feel? (Encourage verbal expression of feelings, both positive and negative, about the past event)

Keep the focus of the session mainly on the process rather than the end result. For example: How did you experience the situation, what positive aspects did you experience? And the negative aspects?

Provide support, encouragement and empathy: Incorporate, as needed, expressions such as: "I understand", "I understand what would happen"...

If necessary, use direct questions to refocus on the event. For example: going back to the event… could you describe how you felt? Or how did you deal with the situation?

Recognize the coping skills used in the event. For example: as you tell me... (Use the words used by the older person to deal with the situation)

It would be positive if everything you are telling me during this week was written in a notebook. Would this be possible? Could do it?

Closing the session: Mr/Ms XXX, we have to end the session, we will continue next week.

**TELEPHONE INTERVENTIONS: OBJECTIVES AND SCRIPT ACCORDING TO SESSION.**

With the elder, identify a theme for the next session (one of the themes that you have identified). On the next call, what topic would you like us to remember? (For the next session, remember about the agreed topic during the week and tell me what you want about the topic. (Narration of the story) and if possible, for next week, could you locate a photo related to the topic to be discussed? (Goal: stimulate memory)

Do you have any questions about what we have been talking about?

Well, next week I will call you again... (Specify the day and time of the telephone session for the following week.)

Record the most important aspects of the session in the field journal.

**Observations:**

**TELEPHONE INTERVENTIONS: OBJECTIVES AND SCRIPT ACCORDING TO SESSION.**

**SESSION 17:** Accompaniment and Reminiscence Therapy.

**Objectives:**

- Keep the bond strong
- Use reminiscence therapy
- Comment on the expression of feelings
- Facilitate the identification of positive aspects of the experience
- Facilitate the identification of negative aspects of the experience
- Facilitate the narration of the lived experience
- Schedule the next call
- Specify the time and date of calls.
- Discuss the expression of both positive and negative aspects of the experience.

**SCRIPT SESSION 17:**

Good afternoon XXXX, this is XXXX

How are you? How has the week been? Any change or situation that has happened to you?

Reminder of the previous session: remember the selected topic to remember. Did you find an object/photo that reminds you of the subject?

We agreed that if you could, to write these memories, have you been able to do it? Would you like to talk about it? (If they have not done so, we agree on another situation to talk about in this session and if they have, we will talk about it). Propose talking about their childhood.

**TELEPHONE INTERVENTIONS: OBJECTIVES AND SCRIPT ACCORDING TO SESSION.**

Tell me, how was your childhood? Facilitate the expression of feelings and the narration of the event. Introduce open-ended questions as needed: What were the three wise men like? Do you remember your parents or your grandparents? ...

How did you feel? (Encourage verbal expression of feelings, both positive and negative, about the past event)

Keep the focus of the session mainly on the process rather than the end result. For example: How did you experience the situation, what positive aspects did you experience? And the negative aspects?

Provide support, encouragement and empathy: Incorporate, as needed, expressions such as: "I understand", "I understand what would happen"...

If necessary, use direct questions to refocus on the event. For example: going back to the event… could you describe how you felt? Or how did you deal with the situation?

Recognize the coping skills used in the event. For example: as you tell me... (Use the words used by the older person to deal with the situation)

It would be positive if everything you are telling me during this week was written in a notebook. Would this be possible? Could do it?

Closing the session: Mr/Ms XXX, we have to end the session, we will continue next week.

**TELEPHONE INTERVENTIONS: OBJECTIVES AND SCRIPT ACCORDING TO SESSION.**

With the elder, identify a theme for the next session (one of the themes that you have identified). On the next call, what topic would you like us to remember? (For the next session, remember about the agreed topic during the week and tell me what you want about the topic. (Narration of the story) and if possible, for next week, could you locate a photo related to the topic to be discussed? (Goal: stimulate memory)

Do you have any questions about what we have been talking about?

Well, next week I will call you again... (Specify the day and time of the telephone session for the following week.)

Record the most important aspects of the session in the field journal.

**Observations:**

**TELEPHONE INTERVENTIONS: OBJECTIVES AND SCRIPT ACCORDING TO SESSION.**

**SESSION 18:** Guidance towards change. Medication and therapeutic adherence, toxic habits and vaccines. Proposal for change.

**Objectives:**

- Keep the bond strong
- Health education: medication
- Health education: toxic habits
- Health education: vaccines
- Proposal for change: go for a walk
- Specify the time and date of calls.

**SCRIPT SESSION 18:**

Good afternoon XXXX, this is XXXX

How are you? How has the week been? Is there anything that you want to tell or ask me?

- If it sounds good to you, in this session we are going to talk about medication. Although we already talked about a few days ago, this is to try to improve habits.

1. Medication- Adherence to therapy.

- Keep medications in the same place: to know at all times what you are taking and avoid interactions and duplication
- Carefully choose where to store the medication: they should always be stored in a cool, dry place, away from children and pets and in some cases in specific places such as the fridge.
- Make an up-to-date list of medications
- Organize the weekly medications
- Check drug interactions
- Make sure the instructions are well understood
- Use medication reminder tools
- Know the possible side effects of each medication

**TELEPHONE INTERVENTIONS: OBJECTIVES AND SCRIPT ACCORDING TO SESSION.**

- Schedule prescription renewals
- Toxic habits (Alcohol and tobacco) (if an older person smokes or drinks, give recommendations for interaction with the medication and help stop smoking or drinking (contact their primary care physician))
- Vaccines (annual flu vaccine, pneumococcal vaccine (between 60-65 years old) 1 dose and at 5 years another until the age of 70, from 70 onwards, only 1 dose without booster and Covid-19 vaccine)

**PROPOSAL FOR CHANGE:**

- We suggest that you go for a walk with a friend, family member, or neighbor and that next week you tell us where you went (a landmark site in your town), with whom, and how you have felt about the walk. If they normally go for a walk alone, suggest that they do it with someone, and if they already do so, invite someone who doesn't normally go for a walk so that they visit the landmark.

Do you have any questions about what we have been talking about?

Well, next week I will call you again... (Specify the day and time of the telephone session for the following week.)

Record the most important aspects of the session in the field journal.

**Observations:**

**TELEPHONE INTERVENTIONS: OBJECTIVES AND SCRIPT ACCORDING TO SESSION.**

**SESSION 19:**

Guidance towards change. Oral hygiene habits, healthy habits: nutrition and hydration and prevention of ulcers and wounds. Maintain proposal for change.

**Objectives:**

- Keep the bond strong
- Health education: oral hygiene habits
- Health education: healthy habits: eating
- Health education: healthy habits, hydration
- Health education: prevention of ulcers and wounds
- Maintain proposal for change.
- Specify the time and date of calls.

**SCRIPT SESSION 19:**

Good afternoon XXXX, this is XXXX

How are you? How has the week been?  Is there anything that you want to tell or ask me?

Last week we suggested that you go for a walk to a landmark place with a relative, friend or neighbor, tell me... Where did you go? Who did you go with? How did you feel? How many days were you out? Propose that they keep going out with someone at least once a week.

If it's okay with you, in this session we're going to talk about diet. Although we already talked about a few days ago, this is to try to improve habits.

1. Oral hygiene habits, food, hydration.

2. Prevention of ulcers and wounds (burns,)

Do you have any questions about what we have been talking about?

**TELEPHONE INTERVENTIONS: OBJECTIVES AND SCRIPT ACCORDING TO SESSION.**

Well, next week I will call you again... (Specify the day and time of the telephone session for the following week.)

Record the most important aspects of the session in the field journal.

**Observations:**

**TELEPHONE INTERVENTIONS: OBJECTIVES AND SCRIPT ACCORDING TO SESSION.**

**SESSION 20:** Guidance towards change. Behavioral habits: constipation and incontinence. Proposal for change and maintain proposal for change.

**Objectives:**

- Maintain the bond
- Health education: behavioral habits, constipation and incontinence.
- Proposal for change: visit associative resource.
- Maintain proposal for change: go for a walk.
- Specify the time and date of calls.

**SCRIPT SESSION 20:**

Good afternoon XXXX, this is XXXX

How are you? How has the week been? Is there anything that you want to tell or ask me?

- Last week we proposed that you keep going out for a walk with someone, tell me... Have you continued doing it? With whom? Where to? How many days?

If it's okay with you, in this session, we are going to talk about incontinence. Although we already talked a few days ago, this is to try to improve habits.

Behavioral habits: constipation and incontinence.

**PROPOSAL FOR CHANGE:**

- Propose that they visit some type of associative resource depending on the interests of the elderly and the town where they live (sports, culture, health and leisure), and that next week they tell us where they have gone and if they have done any activity and what it consisted of.

Do you have any questions about what we have been talking about?

**TELEPHONE INTERVENTIONS: OBJECTIVES AND SCRIPT ACCORDING TO SESSION.**

Well, next week I will call you again... (Specify the day and time of the telephone session for the following week.)

Record the most important aspects of the session in the field journal.

**Observations:**

**TELEPHONE INTERVENTIONS: OBJECTIVES AND SCRIPT ACCORDING TO SESSION.**

**SESSION 21:** Accompaniment and Reminiscence Therapy.

**Objectives:**

- Maintain the bond
- Use reminiscence therapy
- Comment on the expression of feelings
- Facilitate the identification of positive aspects of the experience
- Facilitate the identification of negative aspects of the experience
- Facilitate the narration of the lived experience
- Schedule the next call
- Specify the time and date of calls.
- Identify a significant experience

**SCRIPT SESSION 21:**

Good afternoon XXXX, this is XXXX

How are you? How has the week been? Is there anything that you want to tell or ask me?

IN THIS SESSION WE ARE GOING TO WORK ON THE THINGS YOU HAVE EXPERIENCED IN THE PAST, WHAT DO YOU THINK IF WE TALK ABOUT A TRIP THAT HAS MARKED YOU?

Tell me, how did it happen? Facilitate the expression of feelings and the narration of the event. Introduce open-ended questions as needed: When did it happen? What else do you remember? Who were you with?

**TELEPHONE INTERVENTIONS: OBJECTIVES AND SCRIPT ACCORDING TO SESSION.**

How did you feel? (Encourage verbal expression of feelings, both positive and negative, about the past event)

Keep the focus of the session mainly on the process rather than the end result. For example: How did you experience the situation, what positive aspects did you experience? And the negative aspects?

Provide support, encouragement and empathy: Incorporate, as needed, expressions such as: "I understand", "I understand what would happen"...

If necessary, use direct questions to refocus on the event. For example: going back to the event… could you describe how you felt? Or how did you deal with the situation?

Recognize the coping skills used in the event. For example: as you tell me... (Use the words used by the older person to deal with the situation)

It would be positive if everything you are telling me during this week was written in a notebook. Would this be possible? Could do it?

Closing the session: Mr/Ms XXX, we have to end the session, we will continue next week.

With the elder, identify a theme for the next session (one of the themes that you have identified). On the next call, what topic would you like us to remember? (For the next

session, remember about the agreed topic during the week and tell me what you want about the topic. (Narration of the story) and if possible, for next week, could you locate a photo related to the topic to be discussed? (Goal: stimulate memory)

Do you have any questions about what we have been talking about?

Well, next week I will call you again... (Specify the day and time of the telephone session for the following week.)

**Observations:**

**TELEPHONE INTERVENTIONS: OBJECTIVES AND SCRIPT ACCORDING TO SESSION.**

**SESSION 22:** Accompaniment and Reminiscence Therapy.

**Objectives:**

- Maintain the bond
- Use reminiscence therapy
- Comment on the expression of feelings
- Facilitate the identification of positive aspects of the experience
- Facilitate the identification of negative aspects of the experience
- Facilitate the narration of the experience lived
- Specify the time and date of calls.
- Identify a significant experience

**SCRIPT SESSION 22:**

Good afternoon XXXX, this is XXXX

How are you? How has the week been? Is there anything that you want to tell or ask me?

Do you remember that last week I recommended that you write down the event? Could you do it? Should we discuss it? (If they have not done so, go to the next point)

In this past week, have you thought about any topic to talk about? (If they have thought about it, talk about it and if not, propose the topic)

How about we talk about school?

Tell me, did you go to school? How long did you go for? How were the teachers? What did they do in class? Facilitate the expression of feelings and the narration of the event. Introduce open-ended questions as needed: what else do you remember? Who were you with?

How did you feel? (Encourage verbal expression of feelings, both positive and negative, about the past event)

**TELEPHONE INTERVENTIONS: OBJECTIVES AND SCRIPT ACCORDING TO SESSION.**

Keep the focus of the session mainly on the process rather than the end result. For example: How did you experience the situation, what positive aspects did you experience? And the negative aspects?

Provide support, encouragement and empathy: Incorporate, as needed, expressions such as: "I understand", "I understand what would happen"...

If necessary, use direct questions to refocus on the event. For example: going back to the event… could you describe how you felt? Or how did you deal with the situation?

Recognize the coping skills used in the event. For example: as you tell me... (Use the words used by the older person to deal with the situation)

It would be positive if everything you are telling me during this week was written in a notebook. Would this be possible? Could do it?

Closing the session: Mr/Ms XXX, we have to end the session, next week I will call you again... (Specify the day and time of the telephone session for the following week.)

Record the most important aspects of the session in the field journal.

**Observations:**

## TELEPHONE INTERVENTIONS: OBJECTIVES AND SCRIPT ACCORDING TO SESSION.

**SESSION 23:** Guidance towards change. Healthy habits: activity and exercise, dressing / grooming / toilet and fall prevention. Maintain proposal for change.

**Objectives:**

- Maintain the bond
- Health education: healthy habits: activity/exercise
- Health education: fall prevention
- Education for health: healthy habits: dress/grooming and WC.
- Maintain change proposal
- Specify the time and date of calls.

**SCRIPT SESSION 23:**

Good afternoon XXXX, this is XXXX

How are you? How has the week been?  Is there anything that you want to tell or ask me?

- We suggested that you visit an associative resource, tell me... Where did you go? Who were you with? What did you do? How did you feel?.... suggest that they continue attending, or that they try another resource and that they tell us how it went the following week.

- We also suggested that you continue to go for a walk, tell me... Do you still do it? Who did you go out with?

If it's okay with you, in this session we're going to talk about healthy habits: activity/exercise, dressing/grooming and toilet, fall prevention.

Do you have any questions about what we have been talking about?

**TELEPHONE INTERVENTIONS: OBJECTIVES AND SCRIPT ACCORDING TO SESSION.**

Well, next week I will call you again... (Specify the day and time of the telephone session for the following week.)

Record the most important aspects of the session in the field journal.

Next week a colleague will call you to ask you some questions. Should we specify the day and time of the evaluation call?

**Observations:**

**TELEPHONE INTERVENTIONS: OBJECTIVES AND SCRIPT ACCORDING TO SESSION.**

**SESSION 24:** Guidance towards change. Healthy habits: sleep hygiene. Proposal for change proposal and maintain proposals for change.

**Objectives:**

- Maintain the bond
- Health education: healthy habits: sleep hygiene
- Proposal for change; visit a relative or friend.
- Maintain change proposals.
- Specify the time and date of calls.

**SCRIPT SESSION 24:**

Good afternoon XXXX, this is XXXX

How are you? How has the week been? Is there anything that you want to tell or ask me?

- We suggested that you visit an associative resource, tell me... Where did you go? Who were you with? What did you do? How did you feel?.... suggest that they continue attending, or that they try another resource and that they tell us how it went the following week.

- We also suggested that you continue to go for a walk, tell me... Do you still do it? Who did you go out with?

If that's okay with you, in this session we're going to talk about sleep, healthy habits: sleep hygiene.

Give general sleep hygiene guidelines: respect sleep and wake times, if you take a nap, it should last no more than 30 minutes, avoid stimulants (coffee, tea, soft drinks, chocolate) before bedtime, avoid copious dinners, perform a relaxing routine before bedtime, avoid noise and promote a calm and welcoming environment. If you take medication, take it

half an hour before going to sleep, go for a walk in the morning or in the afternoon, never before going to sleep.

**PROPOSAL FOR CHANGE:**

Propose that they visit a 1st or 2nd degree relative depending on the family network, a close friend in the case of no relatives (cousin, sister-in-law, friend,...) and if they are far away, that they call them by phone and that they tell us the following week.

Do you have any questions about what we have been talking about?

Well, next week I will call you again... (Specify the day and time of the telephone session for the following week.)

Record the most important aspects of the session in the field journal.

**Observations:**

**TELEPHONE INTERVENTIONS: OBJECTIVES AND SCRIPT ACCORDING TO SESSION.**

**SESSION 25:** Accompaniment and Reminiscence Therapy.

**Objectives:**

- Maintain the bond
- Use reminiscence therapy
- Comment on the expression of feelings
- Facilitate the identification of positive aspects of the experience
- Facilitate the identification of negative aspects of the experience
- Facilitate the narration of the experience lived
- Schedule the next call
- Specify the time and date of calls
- Identify a significant experience

**SCRIPT SESSION 25:**

Good afternoon XXXX, this is XXXX

How are you? How has the week been? Is there anything that you want to tell or ask me?

IN THIS SESSION WE ARE GOING TO WORK ON THE THINGS THAT YOU EXPERIENCED IN THE PAST, WHAT DO YOU THINK IF WE TALK ABOUT SINGERS THAT REMINDS YOU OF YOUR YOUTH

Tell me, what kind of music do they sing? Facilitate the expression of feelings and the narration of the event. Introduce open-ended questions as needed: from what period? What song do you like the most? Can you sing it? Do you remember the lyrics? What else do you remember? Who did you listen with? If you listened to the song now, what memories would it bring back?

**TELEPHONE INTERVENTIONS: OBJECTIVES AND SCRIPT ACCORDING TO SESSION.**

Provide support, encouragement and empathy: Incorporate, as needed, expressions such as: "I understand", "I understand what would happen"...

Recognize the coping skills used in the event. For example: as you tell me... (Use the words used by the older person to deal with the situation)

It would be positive if everything you are telling me during this week was written in a notebook. Would this be possible? Could do it?

Closing the session: Mr/Ms XXX, we have to end the session, we will continue next week

With the elder, identify a theme for the next session (one of the themes that you have identified). On the next call, what topic would you like us to remember? (For the next session, remember about the agreed topic during the week and tell me what you want about the topic. (Narration of the story) and if possible, for next week, could you locate a photo related to the topic to be discussed? (Goal: stimulate memory)

Do you have any questions about what we have been talking about?

Well, next week I will call you again... (Specify the day and time of the telephone session for the following week.)

Record the most important aspects of the session in the field journal.

**Observations:**

**TELEPHONE INTERVENTIONS: OBJECTIVES AND SCRIPT ACCORDING TO SESSION.**

**SESSION 26**: Accompaniment and Reminiscence Therapy.

**Objectives:**

- Maintain the bond
- Use reminiscence therapy
- Comment on the expression of feelings
- Facilitate the identification of positive aspects of the experience
- Facilitate the identification of negative aspects of the experience
- Facilitate the narration of the experience lived
- Specify the time and date of calls
- Identify a significant experience

**SCRIPT SESSION 26:**

Good afternoon XXXX, this is XXXX

How are you? How has the week been?  Is there anything that you want to tell or ask me?

Do you remember that last week I recommended that you write down the event? Could you do it? Can we discuss it? (If they have not done so, go to the next point)

Have you thought about any topic about the past that we could talk about this week? (If they have thought about it, talk about it and if not, propose the topic)

What do you think if we talk about your youth, about what parties were like?

**TELEPHONE INTERVENTIONS: OBJECTIVES AND SCRIPT ACCORDING TO SESSION.**

Tell me, how did you have fun in your youth? What fairs or festivals took place in your town? What did you drink? Facilitate the expression of feelings and the narration of the event. Introduce open-ended questions as needed: what else do you remember? who were you with?...

Provide support, encouragement and empathy: Incorporate, as needed, expressions such as: "I understand", "I understand what would happen"...

If necessary, use direct questions to refocus on the event. For example: going back to the event… could you describe how you felt? Or how did you deal with the situation?

Recognize the coping skills used in the event. For example: as you tell me... (Use the words used by the older person to deal with the situation)

Closing the session: Mr/Ms XXX, we have to end the session, next week I will call you again... (Specify the day and time of the telephone session for the following week.)

Record the most important aspects of the session in the field journal.

**Observations:**

**TELEPHONE INTERVENTIONS: OBJECTIVES AND SCRIPT ACCORDING TO SESSION.**

**SESSION 27:** Guidance towards change. Gynecological and urological check-ups. Hygiene measures: UTI prevention. Maintain proposals for change.

**Objectives:**

- Maintain the bond
- Health education: healthy habits: reviews
- Education for health: healthy habits: hygienic measures
- Maintain previous proposals for change
- Specify the time and date of calls.

**SCRIPT SESSION 27:**

Good afternoon XXXX, this is XXXX

How are you? How has the week been? Is there anything that you want to tell or ask me?

- Do you remember that I proposed that you visit or call a relative or friend? Tell me, who did you visit? How did you feel? Propose that next week they call another family member or the same one again.

- We suggested that you visit an associative resource, tell me... Where did you go? Who were you with? What did you do? How did you feel?.... suggest that they continue attending, or that they try another resource and that they tell us how it went the following week.

- Ask if they continue to go out for a walk with a neighbor, friend, family member…. And encourage them to resume it or continue doing it.

If that's okay with you, today we are going to talk about gynecological and urological check-ups, mammography (prostate and cytology) and UTI prevention: hygienic measures.

Do you have any questions about what we have been talking about?

Well, next week I will call you again .... (Specify the day and time of the telephone session for the following week.)

Record the most important aspects of the session in the field journal.

**Observations:**

**TELEPHONE INTERVENTIONS: OBJECTIVES AND SCRIPT ACCORDING TO SESSION.**

**SESSION 28:** Guidance towards change. Social interaction benefits, social resources, general recommendations for abuse and violence, recommendations to avoid primary caregiver overload: available resources (family respite, professional caregivers, home help). Proposal for change and maintain previous proposals for change.

**Objectives:**

- Maintain the bond strong
- Health education: Social interaction benefits
- Health education: social resources
- Health education: mistreatment
- Health education: main caregiver
- Proposal for change: outing or trip
- Maintain previous proposals for change
- Specify the time and date of calls

**SCRIPT SESSION 28:**

Good afternoon XXXX, this is XXXX

How are you? How has the week been? Is there anything that you want to tell or ask me?

Do you remember that I suggested that you visit or call a relative or friend? Tell me, who did you visit? How did you feel? Propose that next week they call another family member or the same one again.

I also suggested that you visit an associative resource, tell me... Where did you go? Who were you with? What did you do? How did you feel?.... suggest that they continue

attending, or that they try another resource and that they tell us how it went the following week.

Ask if they continue to go out for a walk with a neighbor, friend, family member…. And encourage them to resume it or continue doing it.

Benefits of social interaction, social resources, abuse and violence (general recommendations) and recommendations to avoid overloading the main caregiver and explain the resources available (family respite, professional caregivers and home help).

**PROPOSAL FOR CHANGE:**

Propose that they sign up for an excursion or Imserso (Institute of the Elderly and Social Service) trip or from an association.

Do you have any questions about what we have been talking about?

Well, next week I will call you again... (Specify the day and time of the telephone session for the following week.)

Record the most important aspects of the session in the field journal.

**Observations:**

**TELEPHONE INTERVENTIONS: OBJECTIVES AND SCRIPT ACCORDING TO SESSION.**

**SESSION 29:** Guidance towards change. Recommendations to achieve future goals. Maintain proposals for change.

**Objectives:**

- Keep the bond strong
- Health education: achievement of objectives
- Maintain proposals for change.
- Specify the time and date of calls.

**SCRIPT SESSION 29:**

Good afternoon XXXX, this is XXXX

How are you? How has the week been?  Is there anything that you want to tell or ask me?

Do you remember that I suggested that you sign up for an excursion or trip? Tell me, have you been able to look at it? Have you been interested or signed up for any excursion or trip? Propose that if you have not done so, do so.

Do you remember that I proposed that you visit or call a relative or friend? Tell me, who did you visit? How did you feel? Propose that next week they call another family member or the same one again.

Remember that I also suggested that you visit an associative resource, tell me... Where did you go? Who did you go with? What did you do? How did you feel?.... suggest that they continue attending, or that they try another resource and that they tell us how it went the following week.

**<u>TELEPHONE INTERVENTIONS: OBJECTIVES AND SCRIPT ACCORDING TO SESSION.</u>**

Ask if they continue to go out for a walk with a neighbor, friend, family member…. And encourage them to resume it or continue doing it.

Today, if it sounds good to you, we are going to talk about some recommendations to achieve future goals (set a short-term goal, make it realistic, think about positive things,)

Do you have any questions about what we have been talking about?

Well, next week I will call you again... (Specify the day and time of the telephone session for the following week.)

Record the most important aspects of the session in the field journal.

**<u>Observations:</u>**

## TELEPHONE INTERVENTIONS: OBJECTIVES AND SCRIPT ACCORDING TO SESSION.

**SESSION 30:** Accompaniment and Reminiscence Therapy.

**Objectives:**

- Keep the bond strong
- Use reminiscence therapy
- Comment on the expression of feelings
- Facilitate the identification of positive aspects of the experience
- Facilitate the identification of negative aspects of the experience
- Discuss the expression of both positive and negative aspects of the experience.
- Facilitate the narration of the experience lived
- Schedule the next call
- Specify the time and date of calls.
- Identify a significant experience

**SCRIPT SESSION 30:**

Good afternoon XXXX, this is XXXX

How are you? How has the week been? Is there anything that you want to tell or ask me?

IN THIS SESSION WE ARE GOING TO WORK ON THE THINGS YOU HAVE EXPERIENCED IN THE PAST, WHAT DO YOU THINK IF WE TALK ABOUT MOVIES

Tell me, do you have a special movie or one that you have seen many times? Facilitate the expression of feelings and the narration of the event. Introduce open-ended questions as needed: Why do you like to see it? Who are the protagonists? What does the movie remind you of? What else do you remember? Who did you see it with? Where did you see it the first time? If you saw it now, what memories would it bring back?

Provide support, encouragement and empathy: Incorporate, as needed, expressions such as: "I understand", "I understand what would happen"...

**TELEPHONE INTERVENTIONS: OBJECTIVES AND SCRIPT ACCORDING TO SESSION.**

Recognize the coping skills used in the event. For example: as you tell me... (Use the words used by the older person to deal with the situation)

It would be positive if everything you are telling me during this week was written in a notebook. Would this be possible? Could do it?

Closing the session: Mr/Ms XXX, we have to end the session, we will continue next week

With the elder, identify a theme for the next session (one of the themes that you have identified). On the next call, what topic would you like us to remember? (For the next session, remember about the agreed topic during the week and tell me what you want about the topic. (Narration of the story) and if possible, for next week, could you locate a photo related to the topic to be discussed? (Goal: stimulate memory)

Do you have any questions about what we have been talking about?

Well, next week I will call you again... (Specify the day and time of the telephone session for the following week.)

Record the most important aspects of the session in the field journal.

**Observations:**

**TELEPHONE INTERVENTIONS: OBJECTIVES AND SCRIPT ACCORDING TO SESSION.**

**SESSION 31:** Accompaniment and Reminiscence Therapy.

**Objectives:**

- Keep the bond strong
- Use reminiscence therapy
- Comment on the expression of feelings
- Facilitate the identification of positive aspects of the experience
- Facilitate the identification of negative aspects of the experience
- Discuss the expression of both positive and negative aspects of the experience.
- Facilitate the narration of the experience lived
- Specify the time and date of calls.
- Identify a significant experience

**SCRIPT SESSION 31:**

Good afternoon XXXX, this is XXXX

How are you? How has the week been?  Is there anything that you want to tell or ask me?

Do you remember that last week I recommended that you write down the event? Could you do it? Can we discuss it? (If they have not done so, go to the next point)

In the past week, have you thought about any topic about the past to talk about this week? (If they have thought about it, talk about it and if not, propose the topic)

How about we talk about your childhood games?

**TELEPHONE INTERVENTIONS: OBJECTIVES AND SCRIPT ACCORDING TO SESSION.**

Tell me, what did you play? Where did you play or with what? With who? Facilitate the expression of feelings and the narration of the event. Introduce open-ended questions as needed: what else do you remember? Who were you with? Do you still remember any games? Do you have any toys or keep any games?

Provide support, encouragement and empathy: Incorporate, as needed, expressions such as: "I understand", "I understand what would happen"...

If necessary, use direct questions to refocus on the event. For example: going back to the event… could you describe how you felt? Or how did you deal with the situation?

Recognize the coping skills used in the event. For example: as you tell me... (Use the words used by the older person to deal with the situation)

Closing the session: Mr/Ms XXX, we have to end the session, next week I will call you again... (Specify the day and time of the telephone session for the following week.)

Record the most important aspects of the session in the field journal.

**Observations:**

**TELEPHONE INTERVENTIONS: OBJECTIVES AND SCRIPT ACCORDING TO SESSION.**

**SESSION 32:** Guidance towards change. Activity recommendations to improve mood, eye and hearing check-ups, alternative therapies for pain management. Proposals for change and maintain proposals for change.

**Objectives:**

- Keep the bond strong
- Health education: state of mind
- Health education: hearing and vision
- Health education: pain management
- Proposal for change: going to the cinema or theater.
- Maintain previous proposals for change.
- Specify the time and date of calls.

**SCRIPT SESSION 32:**

Good afternoon XXXX, this is XXXX

How are you? How has the week been? Is there anything that you want to tell or ask me?

Do you remember that I proposed that you visit or call a relative or friend? Tell me, who did you visit? How did you feel? Propose that next week they call another family member or the same one again.

Remember that I also suggested that you visit an associative resource, tell me... Where did you go? Who did you go with? What did you do? How did you feel?.... suggest that they continue attending, or that they try another resource and that they tell us how it went the following week.

Ask if they continue to go out for a walk with a neighbor, friend, family member…. And encourage them to resume it or continue doing it.

**TELEPHONE INTERVENTIONS: OBJECTIVES AND SCRIPT ACCORDING TO SESSION.**

Do you remember that I suggested that you sign up for an excursion or trip? Were you able to do it? Where did you go? Did you like it? With whom did you go?

If it's okay with you, today I'm going to give you some activity recommendations to improve your mood (listen to music, go to the movies, and go for a walk (everything that involves leaving the house). If they have depression, recommend that they keep appointments and follow the prescribed treatment.

Also recommend annual check-ups, both ophthalmological and hearing (dry eye, presbyopia, and recommendations for the use of screens…)

Provide information about the use of alternative therapies for pain management (relaxation techniques, music therapy, yoga...) medical and pharmacological follow-up.

**PROPOSAL FOR CHANGE:**

Propose going to the movies or the theater with a relative, friend or neighbor.

Do you have any questions about what we have been talking about?

Well, next week I will call you again... (Specify the day and time of the telephone session for the following week.)

Record the most important aspects of the session in the field journal.

**Observations:**

**TELEPHONE INTERVENTIONS: OBJECTIVES AND SCRIPT ACCORDING TO SESSION.**

**SESSION 33:** Guidance towards change. Relaxation techniques. Offer resources. Change proposal and maintain previous change proposals.

**Objectives:**

- Keep the bond strong
- Health education: relaxation techniques: diaphragmatic breathing
- Health education: response to stressful situations
- Health education: negative thoughts and attitude towards illness
- Maintain previous proposals for change.
- Specify the time and date of calls.

**SCRIPT SESSION 33:**

Good afternoon XXXX, this is XXXX

How are you? How has the week been? Is there anything that you want to tell or ask me?

Do you remember that I proposed that you visit or call a relative or friend? Tell me, who did you visit? How did you feel? Propose that next week they call another family member or the same one again.

Remember that I also suggested that you visit an associative resource, tell me... Where did you go? Who did you go with? What did you do? How did you feel?.... suggest that they continue attending, or that they try another resource and that they tell us how it went the following week.

Ask if they continue to go out for a walk with a neighbor, friend, family member…. And encourage them to resume it or continue doing it.

**TELEPHONE INTERVENTIONS: OBJECTIVES AND SCRIPT ACCORDING TO SESSION.**

Do you remember that I suggested that you sign up for an excursion or trip? Tell me, have you been able to look at it? Have you been interested or signed up for any excursion or trip? Propose that if you have not done so, do so.

Do you remember that I suggested that you go to the movies or the theater? Were you able to do it? Where did you go? Did you like it? Who were you with? What did you see? Suggest that they maintain the activity.

Today, if it's okay with you, we're going to talk about relaxation techniques (diaphragmatic breathing), teach you and practice it.

In today's session, if you like, I am going to inform you about some resources such as: associations for your illness and coping strategies against negative thoughts (visits to the psychologist, referrals from psychological help and Psychologist).

Do you have any questions about what we have been talking about?

Well, next week I will call you again... (Specify the day and time of the telephone session for the following week.)

Record the most important aspects of the session in the field journal.

**Observations:**

**TELEPHONE INTERVENTIONS: OBJECTIVES AND SCRIPT ACCORDING TO SESSION.**

**SESSION 34:** Accompaniment and Reminiscence Therapy.

**Objectives:**

- Keep the bond strong
- Use reminiscence therapy
- Comment on the expression of feelings
- Facilitate the identification of positive aspects of the experience
- Facilitate the identification of negative aspects of the experience
- Discuss the expression of both positive and negative aspects of the experience.
- Facilitate the narration of the lived experience
- Schedule the next call
- Specify the time and date of calls.
- Identify a significant experience

**SCRIPT SESSION 34:**

Good afternoon XXXX, this is XXXX

How are you? How has the week been? Is there anything that you want to tell or ask me?

IN THIS SESSION WE ARE GOING TO WORK ON THE THINGS YOU HAVE EXPERIENCED IN THE PAST, WHAT DO YOU THINK IF WE TALK ABOUT YOUR WORK

Tell me, what kind of work have you done? What did you do? What did you like the most? What did you buy with your first paycheck? Facilitate the expression of feelings and the narration of the event. Introduce open-ended questions as needed: what else do you remember? Who did you work with? How was your retirement? ...

**TELEPHONE INTERVENTIONS: OBJECTIVES AND SCRIPT ACCORDING TO SESSION.**

Provide support, encouragement and empathy: Incorporate, as needed, expressions such as: "I understand", "I understand what would happen"...

Recognize the coping skills used in the event. For example: as you tell me... (Use the words used by the older person to deal with the situation)

It would be positive if everything you are telling me during this week was written in a notebook. Would this be possible? Could do it?

Closing the session: Mr/Ms XXX, we have to end the session, we will continue next week

With the elder, identify a theme for the next session (one of the themes that you have identified). On the next call, what topic would you like us to remember? (For the next session, remember about the agreed topic during the week and tell me what you want about the topic. (Narration of the story) and if possible, for next week, could you locate a photo related to the topic to be discussed? (Goal: stimulate memory)

Do you have any questions about what we have been talking about?

Well, next week I will call you again... (Specify the day and time of the telephone session for the following week.)

Record the most important aspects of the session in the field journal.

**<u>TELEPHONE INTERVENTIONS: OBJECTIVES AND SCRIPT ACCORDING TO SESSION.</u>**

**<u>Observations:</u>**

**SESSION 35:** Accompaniment and Reminiscence Therapy.

**Objectives:**

- Keep the bond strong
- Use reminiscence therapy
- Comment on the expression of feelings
- Facilitate the identification of positive aspects of the experience
- Facilitate the identification of negative aspects of the experience
- Discuss the expression of both positive and negative aspects of the experience.
- Facilitate the narration of the lived experience
- Specify the time and date of calls.
- Identify a significant experience

**SCRIPT SESSION 35:**

Good afternoon XXXX, this is XXXX

How are you? How has the week been? Is there anything that you want to tell or ask me?

Do you remember that last week I recommended that you write down the event? Could you do it? Can we discuss it? (If they have not done so, go to the next point)

This past week, have you thought about any topic about the past to talk? (If they have thought about it, talk about it and if not, propose the topic)

How about if we talk about what your childhood was like with your parents or your first love

**TELEPHONE INTERVENTIONS: OBJECTIVES AND SCRIPT ACCORDING TO SESSION.**

Tell me, what were your parents like? What did they live on? How many siblings were you and how did they take care of each other? Where did they live?

First love, when was it? Did you have chaperones? What did you invite him or her to? What did you do on the first date? When was the first kiss? Did you give her or him anything? Did her or his parents agree or not?

Facilitate the expression of feelings and the narration of the event. Introduce open-ended questions as needed: what else do you remember? Who were you with?

Provide support, encouragement and empathy: Incorporate, as needed, expressions such as: "I understand", "I understand what would happen"...

If necessary, use direct questions to refocus on the event. For example: going back to the event… could you describe how you felt? Or how did you deal with the situation?

Recognize the coping skills used in the event. For example: as you tell me... (Use the words used by the older person to deal with the situation)

Closing the session: Mr/Ms XXX, we have to end the session, next week I will call you again... (Specify the day and time of the telephone session for the following week.)

Next week a colleague will call you to ask you some questions.

Record the most important aspects of the session in the field journal.

**Observations:**

**TELEPHONE INTERVENTIONS: OBJECTIVES AND SCRIPT ACCORDING TO SESSION.**

**SESSION 36:** EVALUATION

**SCRIPT SESSION 36:**

Good afternoon XXXX, this is XXXX

How are you? How has the week been? Is there anything that you want to tell or ask me?

DID YOU LIKE THE CALLS?

DID YOU FEEL COMFORTABLE?

WOULD YOU RECOMMEND THIS STUDY TO MORE PEOPLE IN YOUR SITUATION?

WOULD YOU ADD SOMETHING TO THE CALLS?

DO YOU WANT TO PROPOSE SOMETHING THAT COULD BE IMPROVED?

Do you have any doubts about what we have been talking about these days?

Is there something you want to tell me?

**TELEPHONE INTERVENTIONS: OBJECTIVES AND SCRIPT ACCORDING TO SESSION.**

REVIEW ACTIVE DIAGNOSTICS AND CLOSE THOSE THAT NEED TO BE MODIFIED IN THE PLATFORM

Well, this is the last call we are going to make, I have felt very comfortable talking with you and accompanying you with the memories we have shared, and it has made me grow and learn as a person and as a student. I hope you felt good with me. I recommend that you maintain the guidance activities towards change according to your preferences or the ones you liked the most.

I wish you the best, whatever questions or needs you have, you know you can contact your nurse, who referred you to this study, and I thank you for participating in the study.

Regards,

**Observations:**
