# Supplementary material for: Effects of a Clinical Simulation-Based Training Program for Nursing Students to Address Social Isolation and Loneliness in the Elderly: A Quasi-Experimental Study
Source: Healthcare (Basel). 2023 Sep 19;11(18):2587. doi: 10.3390/healthcare11182587 (PMC10531334; doi:10.3390/healthcare11182587)
Supplement: Supplementary file 1 [file healthcare-11-02587-s001.zip › Table S1 Mario.pdf]

Table S1. Scores obtained in the items from the KAOP questionnaire pre and post training.

Items		Pre-training (*n* = 25)	Post-training (*n* = 25)	Bootstrap analysis §			Pre—Post training		
		M (SD)	*M (SD)*	*M*	*95% CI*		*t*	*p*	*d*
1.	It would probably be much better if seniors lived in residential areas with people their own age*	3.6 (1.15)	5.00 (1.12)	1.40	0.79	2.01	4.76	<0.001	0.95
2.	It would probably be better if seniors lived in residential areas with younger people	4.04 (1.14)	4.76 (0.72)	0.72	0.18	1.26	2.75	0.011	0.55
3.	There is something different in seniors ; it's hard to know if something bothers them a lot*	4.84 (0.99)	5.36 (0.99)	0.52	0.03	1.01	2.18	0.040	0.44
4.	Most seniors are really no different from everyone else; just like younger people, they are easy to understand	3.92 (1.08)	4.84 (0.90)	0.92	0.40	1.44	3.66	0.001	0.73
5.	Most seniors have established ways of being and/or acting that are difficult for them to change*	2.84 (1.03)	4.64 (0.76)	1.80	1.23	2.37	6.50	<0.001	1.30
6.	Most seniors are able to adapt when the situation requires it	3.28 (0.54)	4.32 (0.69)	1.04	0.67	1.41	5.85	<0.001	1.17
7.	Most seniors prefer to retire as soon as their pensions or children can support them.*	3.68 (1.14)	5.40 (0.87)	1.72	1.07	2.37	5.48	<0.001	1.10
8.	Most older people prefer to continue working as long as they can do it rather than depending on someone else	4.40 (0.65)	5.32 (0.99)	0.92	0.37	1.47	3.48	0.002	0.70
9.	Most older people tend to have their homes in an unwanted and unattractive state.*	3.80 (0.58)	5.44 (0.87)	1.64	1.15	2.13	6.91	<0.001	1.38
10.	Most seniors can generally be relied on to maintain a clean and attractive home	3.80 (0.71)	4.76 (0.72)	0.96	0.61	1.31	5.71	<0.001	1.14
11.	It is silly to think that wisdom comes with age.*	3.76 (0.83)	5.08 (0.70)	1.32	0.91	1.73	6.68	<0.001	1.34
12.	La gente se vuelve más sabia con la edad	4.12 (0.78)	5.08 (0.70)	0.96	0.59	1.33	5.40	<0.001	1.08
13.	Older people have a lot of power in business and politics*	3.76 (0.66)	4.56 (0.71)	0.80	0.44	1.16	4.62	<0.001	0.92
14.	Older people should have more power in business and politics	3.72 (0.98)	5.28 (0.84)	1.56	0.99	2.13	5.62	<0.001	1.13
15.	Most old people make you feel uncomfortable.*	3.76 (0.78)	5.36 (1.04)	1.60	1.02	2.18	5.66	<0.001	1.13
16.	Most older people make you feel relaxed when you are around them.	4.00 (0.41)	5.44 (0.87)	1.44	1.03	1.85	7.18	<0.001	1.44
17.	Most older people bore others by insisting on wanting to talk about the "old days"*	4.12 (0.67)	5.56 (0.82)	1.44	0.99	1.89	6.65	<0.001	1.33

18.	One of the most interesting and entertaining qualities of most older people is telling their past experiences	4.32 (0.48)	5.48 (0.82)	1.16	0.72	1.60	5.43	<0.001	1.09
19.	Most older people spend a lot of time meddling in other people's business and giving advice to those who haven't asked for it*	4.00 (0.65)	5.44 (0.87)	1.44	0.99	1.89	6.65	<0.001	1.33
20.	Most older people tend to give advice only when asked.	3.52 (1.08)	5.12 (1.13)	1.60	0.97	2.23	5.24	<0.001	1.05
21.	If older people want other people to like them, their first step should be to get rid of their irritating flaws*	3.84 (0.62)	5.56 (0.82)	1.72	1.27	2.17	7.82	<0.001	1.56
22.	If you think a little, older people have the same flaws as anyone else	3.72 (0.54)	5.40 (0.87)	1.68	1.29	2.07	8.89	<0.001	1.78
23.	In order to have a nice neighborhood, it would be better not to have many older people living there.*	4.20 (0.58)	5.52 (0.82)	1.32	0.82	1.82	5.43	<0.001	1.09
24.	You can count on a nice neighborhood when there are a considerable number of older people living there	3.76 (0.52)	5.44 (0.92)	1.68	1.19	2.17	7.12	<0.001	1.42
25.	There are some exceptions; but in general most older people are very similar*	3.84 (0.55)	5.32 (0.9)	1.48	1.08	1.88	7.69	<0.001	1.54
26.	It is evident that most older people are very different from each other	3.68 (0.75)	5.44 (0.92)	1.76	1.26	2.26	7.33	<0.001	1.47
27.	Most older people should care more about their personal appearance; they are very messy*	3.76 (0.52)	5.44 (0.82)	1.68	1.26	2.10	8.16	<0.001	1.63
28.	Most older people appear quite neat and tidy in their personal appearance.	3.68 (0.56)	5.32 (0.99)	1.64	1.11	2.17	6.37	<0.001	1.27
29.	Most older people are irritable, moody and unpleasant*	4.04 (0.35)	5.48 (0.82)	1.44	1.12	1.76	9.37	<0.001	1.88
30.	Most older people are cheerful, pleasant and in a good mood	3.84 (0.62)	4.76 (0.93)	0.92	0.48	1.36	4.27	<0.001	0.85
31.	Most older people constantly complain about the behavior of the younger generations*	3.16 (0.9)	5.28 (0.89)	2.12	1.56	2.68	7.77	<0.001	1.55
32.	One hardly ever hears older people complain about the behavior of younger generations	2.84 (0.9)	5.00 (1.26)	2.16	1.56	2.76	7.38	<0.001	1.48
33.	Most older people require more love and security than anyone else*	2.28 (1.06)	4.80 (0.50)	2.52	2.00	3.04	9.98	<0.001	2.00

34.	Most older people do not need more love and security than anyone else	2.36 (0.95)	4.52 (0.92)	2.16	1.56	2.76	7.38	<0.001	1.48

§ The Bootstrap results are based on a simulation with 1000 participants

* Negative items

M = man; *SD* = standard deviation; *CI*= Confidence interval; *d* = Cohen's effect size
